# p66ShcA functions as a contextual promoter of breast cancer metastasis

**DOI:** 10.1186/s13058-020-1245-6

**Published:** 2020-01-15

**Authors:** Kyle Lewis, Alex Kiepas, Jesse Hudson, Julien Senecal, Jacqueline R. Ha, Elena Voorand, Matthew G. Annis, Valerie Sabourin, Ryuhjin Ahn, Rachel La Selva, Sébastien Tabariès, Brian E. Hsu, Matthew J. Siegel, Matthew Dankner, Eduardo Cepeda Canedo, Mathieu Lajoie, Ian R. Watson, Claire M. Brown, Peter M. Siegel, Josie Ursini-Siegel

**Affiliations:** 1 Lady Davis Institute for Medical Research, 3755 Chemin de la Côte-Sainte-Catherine, Montreal, QC H3T 1E2 Canada; 2 Department of Biochemistry, McGill University, 3655 Promenade Sir William Osler, Montreal, QC H3G 1Y6 Canada; 3 Department of Physiology, McGill University, 3655 Promenade Sir William Osler, Montreal, QC H3G 1Y6 Canada; 4 Goodman Cancer Research Centre, McGill University, 1160 Pine Avenue, West, Room 513, Montreal, QC H3A 1A3 Canada; 5 Division of Experimental Medicine, McGill University, 1001 Decarie Boulevard, Montreal, QC H4A 3J1 Canada; 6 Department of Medicine, McGill University, 1001 Decarie Boulevard, Montreal, QC H3G 1Y6 Canada; 7 Gerald Bronfman Department of Oncology, McGill University, 5100 Maisonneuve Blvd West, Montreal, QC H4A 3T2 Canada

**Keywords:** Breast cancer, Lung metastasis, p66ShcA, Reactive oxygen species

## Abstract

**Background:**

The p66ShcA redox protein is the longest isoform of the *Shc1* gene and is variably expressed in breast cancers. In response to a variety of stress stimuli, p66ShcA becomes phosphorylated on serine 36, which allows it to translocate from the cytoplasm to the mitochondria where it stimulates the formation of reactive oxygen species (ROS). Conflicting studies suggest both pro- and anti-tumorigenic functions for p66ShcA, which prompted us to examine the contribution of tumor cell-intrinsic functions of p66ShcA during breast cancer metastasis.

**Methods:**

We tested whether p66ShcA impacts the lung-metastatic ability of breast cancer cells. Breast cancer cells characteristic of the ErbB2+/luminal (NIC) or basal (4T1) subtypes were engineered to overexpress p66ShcA. In addition, lung-metastatic 4T1 variants (4T1-537) were engineered to lack endogenous p66ShcA via Crispr/Cas9 genomic editing. p66ShcA null cells were then reconstituted with wild-type p66ShcA or a mutant (S36A) that cannot translocate to the mitochondria, thereby lacking the ability to stimulate mitochondrial-dependent ROS production. These cells were tested for their ability to form spontaneous metastases from the primary site or seed and colonize the lung in experimental (tail vein) metastasis assays. These cells were further characterized with respect to their migration rates, focal adhesion dynamics, and resistance to anoikis in vitro. Finally, their ability to survive in circulation and seed the lungs of mice was assessed in vivo.

**Results:**

We show that p66ShcA increases the lung-metastatic potential of breast cancer cells by augmenting their ability to navigate each stage of the metastatic cascade. A non-phosphorylatable p66ShcA-S36A mutant, which cannot translocate to the mitochondria, still potentiated breast cancer cell migration, lung colonization, and growth of secondary lung metastases. However, breast cancer cell survival in the circulation uniquely required an intact p66ShcA S36 phosphorylation site.

**Conclusion:**

This study provides the first evidence that both mitochondrial and non-mitochondrial p66ShcA pools collaborate in breast cancer cells to promote their maximal metastatic fitness.

## Background

The *ShcA* gene encodes three isoforms (p46, p52, and p66), which together integrate mitogenic and oxidative stress responses to dynamically regulate cell fate decisions (as reviewed in [1–4]). p46/p52ShcA are encoded from a single transcript and arise through alternate translational start sites [5]. In contrast, p66ShcA is more variably expressed and encoded by its own promoter [6]. ShcA isoforms exert diverse biological functions. Whereas p46/p52ShcA transduce mitogenic signals [4, 5], p66ShcA induces oxidative stress by facilitating mitochondrial-dependent reactive oxygen species (ROS) production [7].

ShcA isoforms share an amino-terminal phospho-tyrosine-binding (PTB) domain, a carboxy-terminal Src-homology 2 (SH2) domain, and a central collagen-homology 1 (CH1 domain) harboring three tyrosine phosphorylation sites [4]. However, p66ShcA uniquely possesses a CH2 domain at its amino terminus, containing a serine residue (S36) that is essential for its biological function as a redox protein. Phosphorylation of S36 by stress kinases permits binding of the Pin1 prolyl isomerase, facilitating p66ShcA mitochondrial translocation [8, 9]. In the mitochondria, p66ShcA stimulates ROS production by binding to cytochrome c and facilitating the transfer of electrons from cytochrome c to molecular oxygen [10].

The role of p66ShcA in cancer development is complex and context dependent. Both mitochondrial and non-mitochondrial p66ShcA pools influence cancer progression, and the variability in how p66ShcA influences cancer cells is consistent with the fact that ROS functions as a double-edged sword in cancer [11, 12]. In lung cancer, increased p66ShcA levels are associated with improved patient outcome [13]. Aggressive lung cancers upregulate Aiolos, a lymphocyte-lineage restricted transcription factor that epigenetically silences p66ShcA [13]. In addition, p66ShcA reduced the metastatic potential of lung cancers in mouse models [14]. The tumor-suppressive properties of p66ShcA in lung cancer are associated with several mechanisms. For example, p66ShcA restrains Ras signaling in lung cancer cells by reducing activation of Grb2/SOS signaling complexes [6, 14]. In addition, p66ShcA suppresses an epithelial-to-mesenchymal transition (EMT) in lung cancer cells [15] and increases anoikis [16, 17].

Paradoxically, p66ShcA largely confers pro-tumorigenic properties in breast, ovarian, and prostate cancers. p66ShcA is overexpressed in each of these cancers compared to benign tissue [18–20]. In breast cancer, independent studies provide opposing data regarding the relationship between p66ShcA levels and patient outcome. In one study, breast tumors with elevated p66ShcA levels combined with reduced tyrosine phosphorylation of the p46/52 ShcA isoforms were associated with good outcome [21]. However, an independent study showed that p66ShcA is overexpressed in breast cancer cell lines and primary tumors with increasing metastatic properties [18].

Multiple mechanisms may explain the increased tumorigenic potential associated with p66ShcA in these cancers. For example, p66ShcA overexpression increases the proliferative rate of ovarian and prostate cancers [20, 22]. Moreover, p66ShcA increases the migratory properties of prostate and breast cancer cells [1, 23, 24] by its recruitment to focal adhesion complexes, thereby regulating Rac1-mediated actin remodeling [16, 25]. Furthermore, p66ShcA activates the Arf6 monomeric G protein in breast cancer cells to potentiate Ras signaling [26]. We recently demonstrated that p66ShcA induces an EMT in breast cancer cells [23]. Finally, a unique role for p66ShcA in hypoxia survival and the acquisition of stem-like features has been described in breast cancer cells [27].

We established a fundamental role for the p46/52ShcA isoforms in breast cancer progression to metastatic disease [28, 29]; however, the precise role of p66ShcA in breast cancer metastasis remains poorly defined. Indeed, p66ShcA plays a complex role in transducing biomechanical signals that promote anchorage-dependent proliferation of cancer cells while paradoxically increasing anoikis following matrix detachment [30]. In this study, we explore the biological significance of p66ShcA during breast cancer metastasis.

## Methods

### Mouse tumor grafts and resections for spontaneous metastasis

All animal studies were approved by the Animal Resources Centre at McGill University and complied with guidelines set by the Canadian Council of Animal Care. Orthotopic injections into the mammary fat pads were performed in BALB/c mice for 4T1-derived cell populations and FVB mice for NIC cell lines. Breast cancer cells were injected at a 1:1 mixture with Matrigel. Tumor size was determined by caliper measurements of two dimensions and volume calculated as follows: 4/3π × width × length^2^ (smaller measurement is always width). Tumors were resected once they reached 500 mm^3^.

### Metastasis assays

The lung-metastatic 4T1 derivatives (537 cell population) were injected into the tail veins of BALB/c mice. Mice were examined daily for signs of respiratory distress, and after the indicated time periods, cohorts were euthanized, and lungs subjected to histological and IHC analysis. For lung colonization assays, the indicated cell populations were stained in vitro with 1 μM of Cell Tracker Red CMPTX dye (Cat. #: C34552, Invitrogen) for 45 min in serum-free media. The cells were then washed twice in PBS prior to tail vein injection. The lungs were removed at 1 h or 24 h post-injection, and the left lobe from each animal was whole-mounted for imaging. Images were captured on the Zeiss Axiozoom.V16 microscope at × 50 magnification using 5 fields of view to cover the lung area. The auto-fluorescence in the green channel was used to obtain a topography image of the lung area. Cells were quantified using ImageJ by applying an Otsu threshold and scoring the number of signals with a square pixel size larger than 0.001.

### Live-cell tracking

Cells were seeded onto μ-slide 8 well coverslips (Cat. #: 80821, ibidi), at a density of 1500 cells/cm^2^, approximately 16–24 h prior to imaging. Coverslips were coated with human plasma fibronectin diluted to a concentration of 5 μg/cm^2^ with 1× PBS for 1 h at 37 °C. Images were acquired in phase contrast every 10 min for 24 h on a Zeiss AxioObserver fully automated inverted microscope equipped with a Plan-Neofluar 10x/0.3NA Ph1 objective, Axiocam 506 camera (Carl Zeiss, Jena, Germany), and Chamlide TC-L-Z003 top-stage incubator system (Live Cell Instrument, Seoul, South Korea). Cells were then manually tracked using MetaXpress analysis software (v. 5.3.0.5; Molecular Devices, Sunnyvale, CA). X, Y position data for each cell track was exported to MATLAB (v. 8.6.0, rel 2015b; The MathWorks, Natick, MA) for further analysis. Rose plots depicting cell movement were created by superimposing the starting position of each cell track on the origin (0, 0). The average speed of each cell was calculated by determining the mean distance traveled between each time point over the imaging interval (10 min). A histogram of cell speed was created in Prism 7 (v. 7.0a; GraphPad Software, La Jolla, CA) by binning data into 5 μm/h intervals ranging from 10 to 70 μm/h. The data was subsequently smoothed using a spline curve and plotted against relative frequency.

### Imaging cellular adhesions

Cells were seeded onto 35-mm cover-glass bottom cell culture dishes (Cat. #: FD35-100, World Precision Instruments) and transfected with 1 μg of pmCherry paxillin (Cat. #: 50526, Addgene). Media were changed 18–24 h after transfection, and cells were allowed to recover for 24 h. Images were acquired every 20 s for 20 min on a Total Internal Reflection Fluorescence (TIRF)-Spinning Disk Spectral Diskovery System (Spectral Applied Research, Richmond Hill, ON) based on a Leica DMI 6000 microscope stand (Quorum Technologies, Puslinch, ON) equipped with a Leica Plan-Apochromat 63x/1.47NA oil DIC objective, ImagEM X2 EM-CCD camera (Hamamatsu Photonics K.K., Hamamatsu City, Japan), and Chamlide CU-501 top-stage incubator system (Live Cell Instrument, Seoul, South Korea). Each cell was illuminated with a 561-nm diode laser set to 22.7% (or ~ 74 μW power). An ET600/50m emission filter (Chroma, Bellows Falls, VT) was used to capture mCherry fluorescence. The camera exposure time was set to 500 ms with an EM gain of 255 and read speed of 22 MHz. A total internal reflection fluorescence (TIRF) prism was used to limit fluorescence excitation to a depth of 80 nm.

### Measuring adhesion dynamics

Images collected with the TIRF microscope were processed in Imaris (v. 8.3.1; Bitplane, Zurich, Switzerland) using the Surfaces function. Briefly, the leading edge of each cell was manually selected using the region of interest tool. Surface detail was smoothed and set to 0.300 μm with a local background subtraction of 0.300 μm. Adhesions were then masked by adjusting the threshold and splitting touching objects with a seed point diameter of 0.700 μm. Finally, adhesions were tracked over time using an autoregressive algorithm with a max distance of 2 μm and gap size of 3 time points. Surfaces smaller than 10 voxels and tracks shorter than 2 min (or 6 time points) were removed with filters.

Mean intensity data for each adhesion was then exported to MATLAB for further analysis. In the first part of the algorithm, a spline curve was fitted to each intensity trace to identify segments of assembly and disassembly. The difference in intensity between each time point was calculated and changes greater than 20% were considered significant. A string of 5 or more upward points was interpreted as assembly, while 5 or more downward points were interpreted as disassembly. In the second part of the algorithm, a log-linear fitting method was used to determine the rate for each event. Fits with an *R*^2^ value greater than 0.7 were significant. Assembly and disassembly rates from three independent experiments were combined to determine the mean for each condition.

### Characterizing adhesion size and aspect ratio

A representative image of each analyzed cell for adhesion dynamics was chosen to be re-processed in Imaris. Surface detail was smoothed and set to 0.200 μm with a local background subtraction of 0.200 μm. All adhesions within a cell were masked by adjusting the threshold and splitting touching objects with a seed point diameter of 0.700 μm. Surfaces smaller than 10 voxels were removed with filters. The semi-minor and semi-major axis lengths for each adhesion were then compared in MATLAB to determine aspect ratio. The data shown represents the mean ± SEM for all cells analyzed in three independent experiments. Similarly, area data for each adhesion was exported from Imaris into Prism 7 to determine mean ± SEM for all cells analyzed in three independent experiments. A histogram of adhesion size was generated by binning data into 1-μm intervals ranging from 0 to 8.5 μm. A line plot for p66-CR (VC) was subsequently added to emphasize the change in distribution. For further information regarding experimental procedures, please see the supplemental materials and methods (Additional file 13).

## Results

### p66ShcA is not sufficient to increase the lung-metastatic potential of breast cancer cells

**Fig. 1:**
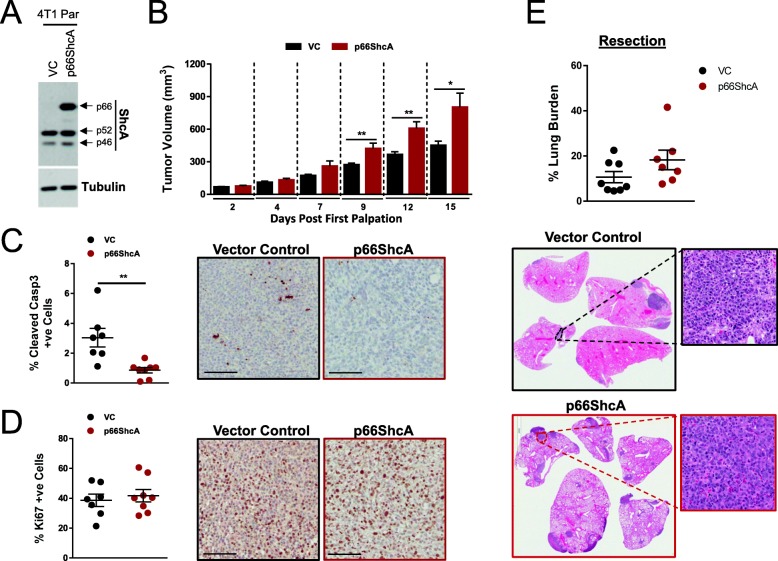
p66ShcA expression is not sufficient to enhance triple-negative breast cancer lung metastasis. **a** Immunoblot analysis of ShcA in vector control (VC) and p66ShcA-overexpressing parental 4T1 breast cancer cells. An immunoblot for α-Tubulin served as a loading control. **b** Mammary fat pad (MFP) injections of VC and p66ShcA-overexpressing parental 4T1 cells. The data is shown as average tumor volume (mm^3^) ± SEM (*n* = 13 tumors/group). **c** Cleaved caspase-3 IHC of VC and p66ShcA-overexpressing 4T1 mammary tumors. The data is shown as average percentage of positive cells ±SEM (*n* = 8 tumors/group). Representative images are shown. **d** Ki67 IHC staining of VC and p66ShcA-overexpressing 4T1 mammary tumors. The data is shown as the average percentage of positive cells ±SEM (*n* = 8 tumors/group). Representative images are shown. **e** Metastatic tumor burden in the lungs of mice bearing VC- and p66ShcA-overexpressing 4T1 mammary tumors. Mammary tumors were resected at 500 mm^3^, and the development of lung metastases was quantified 14 days later. The data is shown as the average percentage of lung metastasis area to lung tissue area ± SEM (*n* = 8 tumors/group). Representative H&E images are shown. Statistical analysis was performed using a two-tailed unpaired Student’s *t* test (*P < 0.05; **P < 0.01)

To determine the impact of p66ShcA on breast cancer metastasis, we chose established models of ErbB2+/Luminal (NIC) [29] and triple-negative (4T1) [31] disease. Both cell models are syngeneic in immunocompetent mice and express low levels of endogenous p66ShcA (Fig. 1a, Additional file 1: Figure S1A). We overexpressed p66ShcA in both cell lines and confirm that increased p66ShcA levels do not alter expression of the other ShcA isoforms (Fig. 1a, Additional file 1: Figure S1A). To examine the effects of p66ShcA on mammary tumor growth, vector control (VC) and p66ShcA-overexpressing cells were injected in the mammary fat pads of FVB (NIC) or Balb/c (4T1) mice. We observe cell context-specific effects of p66ShcA on tumor growth in both models. Whereas p66ShcA increased the tumorigenic potential of 4T1 breast cancer cells compared to VC cells, p66ShcA-expressing NIC tumors displayed reduced mammary tumor growth. (Fig. 1b, Additional file 1: S1B). The increased growth potential of p66ShcA-overexpressing 4T1 tumors is attributed to a decreased apoptotic rate (as assessed by cleaved caspase-3 immunohistochemistry (IHC)) (Fig. 1c). In contrast, there is no difference in the proliferative ability of VC and p66ShcA-expressing 4T1 tumors, as assessed by Ki67 IHC staining (Fig. 1d). The impaired growth potential of NIC/p66ShcA mammary tumors is largely attributed to reduced cell cycle progression with little impact on the degree of apoptosis, compared to vector controls (Additional file 1: Figure S1C, D). These data suggest that p66ShcA can either inhibit or promote breast tumor growth, likely depending on genetic, epigenetic, or post-translational mechanisms contributing to breast tumor heterogeneity.

To assess the impact of p66ShcA on lung metastasis, a separate experiment was performed in which primary tumors were resected at a tumor volume of 500 mm^3^ and lung-metastatic burden quantified 14 days (4T1) or 21 days (NIC) post-resection. We observed no significant differences in the lung-metastatic burden between mice bearing VC- and p66ShcA-expressing mammary tumors in either the NIC or 4T1 models (Fig. 1e, Additional file 1: Figure S1E). These findings indicate that p66ShcA overexpression is not sufficient to increase breast cancer lung metastasis.

### p66ShcA is required for breast cancer lung metastasis

These findings do not preclude the possibility that p66ShcA may be required for dissemination of breast cancer cells that have already acquired increased lung-metastatic potential. We examined endogenous p66ShcA expression levels in cell populations that were in vivo selected to aggressively metastasize to the lung, liver, or bone [32–34], three common metastatic sites for human breast cancer. Breast cancer cell lines established following in vivo passage through the mammary fat pad served as negative controls. Endogenous p66ShcA levels were increased in lung- and liver-metastatic breast cancer populations relative to explants that were in vivo passaged through the bone or mammary fat pad (Additional file 2: Figure S2A). Moreover, 33% (12/36) of single cell clones derived from parental 4T1 cells exhibit elevated endogenous p66ShcA levels, suggesting that these cell populations pre-existed in the tumor and were specifically enriched in breast cancer cells that metastasized to the lung or liver (Additional file 2: Figure S2B). This is consistent with our interrogation of publically available datasets showing that metastases isolated from breast cancer patients displayed significantly higher *p66ShcA* mRNA levels compared to primary breast tumors (Additional file 2: Figure S2C). Therefore, we explored whether p66ShcA was necessary for the increased formation of breast cancer lung metastases by stably deleting p66ShcA from a lung-metastatic 4T1 variant (4T1-537) using CRISPR/Cas9 approaches. Individual p66ShcA null clones were pooled to generate a polyclonal cell population (Additional file 2: Figure S2D, E).

**Fig. 2:**
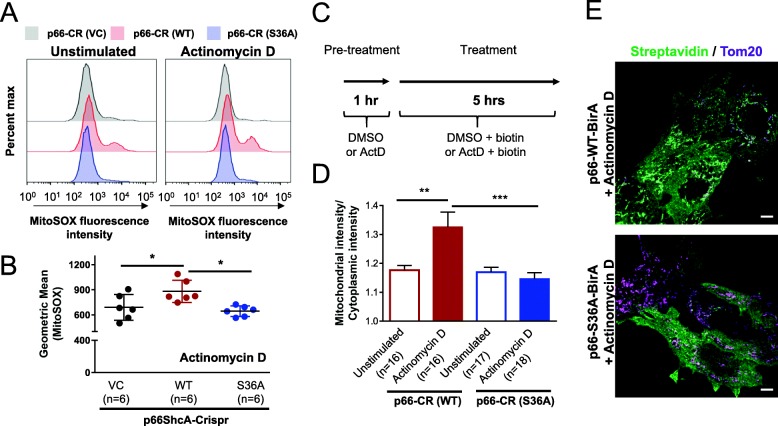
Non-mitochondrial p66ShcA pools are retained in the cytoplasm and impaired in mitochondrial ROS production. **a** Wild-type p66ShcA, but not the p66ShcA^S36A^ mutant, induces ROS production following translocation to the mitochondria. Representative flow cytometry histograms of MitoSOX Red fluorescence in p66-CR (VC), p66-CR (WT), and p66-CR (S36A)-expressing 537-4T1 lung-metastatic breast cancer cells following a 2-h treatment with DMSO (unstimulated) or Actinomycin D. **b** Geometric mean of MitoSOX intensity for p66-CR (VC), p66-CR (WT), and p66-CR (S36A)-expressing 537-4T1 breast cancer cells treated with Actinomycin D. Each point depicts an independent experiment (*n* = 6 total). **c** Schematic depiction of the treatment protocol applied to p66-WT-BirA or p66-S36A-BirA expressing 537-4T1 lung-metastatic breast cancer cells prior to immunofluorescence staining to detect biotinylated proteins. **d** Quantification of streptavidin immunofluorescence intensity (biotinylated proteins) within mitochondria normalized to streptavidin immunofluorescence in the cytoplasm following treatment with DMSO (unstimulated) or Actinomycin D. **e** Representative merged images of streptavidin and Tom20 localization in p66-WT-BirA and p66-S36A-BirA expressing 537-4T1 breast cancer cells treated with Actinomycin D. Scale bar is 5 μm. For panel **b**, statistical analysis was performed using a one-way ANOVA with a Tukey’s multiple comparisons test (*P < 0.05). For panel **d**, statistical analysis was performed using a two-way ANOVA with a Tukey’s multiple comparisons test (**P < 0.01; ***P < 0.001)

The ability of p66ShcA to translocate to the mitochondria and stimulate ROS formation requires S36 phosphorylation [7]. To assess the contributions of mitochondrial and non-mitochondrial p66ShcA pools on breast cancer growth and metastasis, we re-expressed versions of p66ShcA that can [p66-CR (WT)] or cannot [p66-CR (S36A)] generate mitochondrial ROS in p66-null [p66-CR (VC)] lung-metastatic breast cancer cells. Immunoblot analysis revealed stable loss of endogenous p66ShcA expression in the vector control [p66-CR (VC)] cells and equivalent p66ShcA levels in the p66-CR (WT)- and p66-CR (S36A)-expressing cells (Additional file 2: Figure S2E). We further demonstrate that p66ShcA is phosphorylated on Ser36 under basal conditions in 4T1-537 cells that re-express wild-type p66ShcA but not the S36A mutant (Additional file 3: Figure S3A). Moreover, stimulation of p66-CR (WT) cells with phenformin or arsenic, both of which stimulate ROS production, further potentiated Ser36 phosphorylation of p66ShcA (Additional file 3: Figure S3A, B). We next examined whether wild-type p66ShcA or the S36A mutant differed in their ability to stimulate mitochondrial-dependent ROS production in 4T1-537 breast cancer cells using MitoSOX flow cytometric analysis. Whereas p66ShcA modestly increased mitochondrial ROS levels under basal conditions, we observed a selective and significant increase in mitochondrial superoxide anion production in wild-type p66ShcA-expressing cells in response to a ROS-inducing chemotherapy (actinomycin D) (Fig. 2a, b). In contrast, the non-phosphorylatable p66ShcA mutant (S36A) showed mitochondrial ROS levels that were comparable to p66ShcA-null cells (Fig. 2a, b). Finally, we also characterized the ability of wild-type p66ShcA or the S36A mutant to translocate into the mitochondria in response to stress stimuli. To test this, either p66ShcA-WT or p66ShcA-S36A constructs were fused to BirA, allowing p66ShcA itself or p66ShcA-interacting proteins to be biotinylated. Breast cancer cells were pre-treated for 1 h with actinomycin D to stimulate oxidative stress and subsequently incubated for 5 h with biotin in the continued presence of actinomycin D or vehicle control (DMSO) (Fig. 2c). Biotinylated p66ShcA was visualized by confocal microscopy using fluorescently conjugated streptavidin antibodies. The degree of mitochondrial co-localization was determined using Tom20-specific antibodies. Quantification of the streptavidin fluorescence intensity in mitochondria (co-localization with Tom20) versus the cytoplasm revealed that only p66ShcA (WT)-BirA proteins were able to translocate into the mitochondria in response to actinomycin D (Fig. 2d, e; Additional file 4: Figure S4). In contrast, p66ShcA (S36A)-BirA molecules were largely restricted to the cytoplasm under similar conditions (Fig. 2d, e; Additional file 4: Figure S4). Taken together, these observations confirm that only lung-metastatic breast cancer cells expressing wild-type p66ShcA, and not the S36A mutant, show increased S36 phosphorylation as well as p66ShcA mitochondria translocation and ROS production in response to inducers of oxidative stress.

**Fig. 3:**
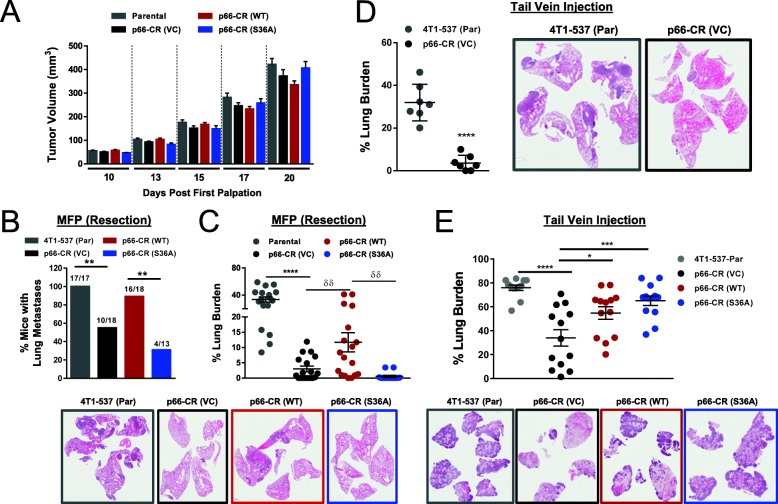
p66ShcA is required for efficient triple-negative breast cancer lung metastasis. **a** Mammary fat pad (MFP) injection of parental, p66-CR (VC), p66-CR (WT), and p66-CR (S36A) expressing lung-metastatic 4T1 cells (537 population). The data is shown as average tumor volume (mm^3^) ± SEM (*n* = 18 tumors/group). **b** Percentage of mice with lung metastases following primary tumor resection. Mice were sacrificed 21 days post-resection of the primary tumor. Statistical analysis was performed using a Fisher’s exact test (**P < 0.01). **c** Metastatic burden, following primary tumor resection, in the lungs of mice bearing the indicated 537 breast cancer cell populations. The data is shown as average lung tumor burden ±SEM (parental: *n* = 17; p66-CR (VC): *n* = 18; p66-CR (WT): *n* = 18; p66-CR (S36A): *n* = 13). Representative H&E images are shown. **d** Metastatic burden in the lungs of mice following tail vein injection of the indicated 537 lung-metastatic breast cancer cells. All mice were necropsied 21 days post-injection. The data is shown as average lung tumor burden ±SEM (*n* = 7 mice per cohort). Representative H&E images are shown. **e** Metastatic burden in the lungs of mice following tail vein injection of the indicated 537 lung-metastatic breast cancer cells. Mice bearing parental 537 cells were necropsied 21 days post-injection, whereas the remaining mice were necropsied 26 days post-injection. Representative H&E images are shown. The data is shown as average lung tumor burden ±SEM (*n* = 13 mice per cohort). For panel **b**, statistical analysis was performed using a Fisher’s exact test (**P < 0.01). For panels **c**–**e**, statistical analysis was performed using a one-way ANOVA with a Tukey’s multiple comparison test (*P < 0.05; **P < 0.01; ***P < 0.001)

To assess the impact of p66ShcA expression and Ser36 phosphorylation on breast cancer growth and metastasis, we injected 4T1-537 (Parental), p66-CR (VC), CR (WT), and CR (S36A) cell populations into the mammary fat pads of mice. These data revealed no appreciable differences on primary tumor growth between any of the cell lines tested (Fig. 3a). We further show that relative p66ShcA levels are comparable between p66-CR (WT) and p66-CR (S36A) mammary tumors (Additional file 5: Figure S5). Moreover, we observed similar proliferative and apoptotic rates of mammary tumors (Additional file 6: Figure S6A, B). Thus, p66ShcA is dispensable for the growth of breast tumors that already acquired more aggressive properties. We next asked whether p66ShcA was necessary for the increased lung-metastatic potential of more aggressive breast tumors. Following surgical resection, we observed a significant reduction in the incidence of lung metastasis in mice bearing p66ShcA-null tumors compared to p66ShcA-proficient controls (537-Par: 100% incidence; p66-CR (VC): 56% incidence) (Fig. 3b). Moreover, p66ShcA deficiency in mammary tumors resulted in a ~ 10-fold reduction in the lung-metastatic burden compared to parental controls (Fig. 3c). These results suggest that p66ShcA is necessary to increase the lung-metastatic potential of mammary tumors that have adapted to elevated p66ShcA levels. We further showed that ectopic re-expression of p66ShcA into p66ShcA-null tumors almost rescued the ability of mammary tumors to metastasize to the lung (89% incidence of lung metastases) (Fig. 3b). In addition, we observed a partial rescue (~ 4-fold) of the lung-metastatic burden in p66-CR (WT) breast cancer cells compared to p66ShcA-deficient controls (Fig. 3c). Finally, reconstitution with the p66ShcA-S36A mutant further debilitated the lung-metastatic potential of p66ShcA-null mammary tumors (p66-CR (VC): 56% incidence; p66-CR (S36A): 31% incidence) (Fig. 3b), coincident with a 5-fold reduction in metastatic burden (Fig. 3c). Combined, these data suggest that p66ShcA Ser36 phosphorylation is required to potentiate spontaneous breast cancer lung metastasis.

### Non-mitochondrial p66ShcA pools potentiate the later stages of the metastatic cascade

To better define the steps of the metastatic cascade that are influenced by p66ShcA, breast cancer cells were injected directly into bloodstream via the lateral tail vein. An initial experiment comparing parental 537 cells with p66-CR (VC) cells employed a single endpoint (21 days) at which time mice were analyzed for lung-metastatic burden. Loss of p66ShcA [p66-CR (VC)] negatively impacted the metastatic ability of breast tumors, as revealed by a 10-fold reduction in the lung-metastatic burden relative to parental cells (Fig. 3d). Given the aggressive nature of 4T1-537 cells, we wished to distinguish between effects due to inefficient lung colonization versus impaired outgrowth to form macroscopic metastases. To test this, we performed a second experimental metastasis assay whereby mice injected with parental 4T1-537 cells were sacrificed early (21 days) while mice injected with p66-CR (VC), p66-CR (WT), or p66-CR (S36A) cells were followed for longer prior to sacrifice (26 days). Again, we observed a reduced metastatic burden (~ 2.2-fold) in mice injected with p66-CR (VC) cells compared to 4T1-537 controls (Fig. 3e). Surprisingly, both p66CR (WT)- and p66-CR (S36A)-expressing cells rescued the metastatic ability of p66ShcA-null cells (Fig. 3e). Combined, these data suggest that p66ShcA is essential for the early (prior to intravasation) and late (following intravasation) stages in the metastatic cascade. Our data further suggests that mitochondrial-localized p66ShcA is required either before, during, or immediately following intravasation, whereas non-mitochondrial p66ShcA pools potentiate extravasation, colonization, and/or secondary growth of breast cancer lung metastases.

### p66ShcA supports efficient breast cancer lung metastasis independently of an EMT

We examined whether the ability of p66ShcA to induce an EMT was associated with an increased lung-metastatic potential. Consistent with our published studies [23], p66ShcA increases Vimentin expression in ErbB2+ luminal breast tumors (NIC) whereas E-Cadherin levels are largely unaffected compared to control tumors (Additional file 7: Figure S7). Despite this fact, we observed no differences in the lung-metastatic potential of VC- or p66ShcA-expressing NIC tumors (Additional file 1: Figure S1E). Moreover, whereas p66ShcA-S36A expressing tumors were significantly debilitated in their lung-metastatic potential, they showed a profound increase in Vimentin levels and a corresponding reduction in E-Cadherin expression (Additional file 7: Figure S7). These data suggest that although non-mitochondrial p66ShcA pools increase the mesenchymal properties of luminal breast cancers, the ability of p66ShcA to induce an EMT is not enough to promote breast cancer lung metastasis. We also examined E-Cadherin and Vimentin levels in 4T1-537 parental, p66-CR (VC), p66-CR (WT), and p66-CR (S36A) mammary tumors, which require p66ShcA for spontaneous breast cancer lung metastasis. We do not observe appreciable differences in Vimentin or E-Cadherin levels in any of these tumors (Additional file 5: Figure S5A, D, E). Thus, p66ShcA supports breast cancer lung metastasis in this TNBC model, even in the absence of an EMT.

### Non-mitochondrial p66ShcA pools enhance breast cancer cell migration by promoting adhesion dynamics

**Fig. 4:**
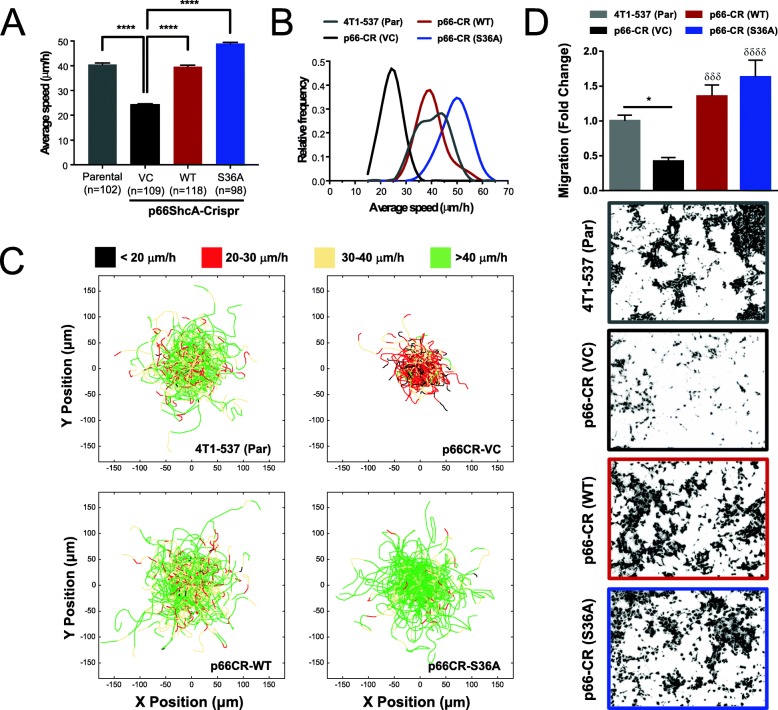
Non-mitochondrial functions of p66ShcA support breast cancer cell migration. **a** Average migration speed of the indicated cell lines was determined by live-cell imaging. The data is representative of average speed (μm/h) ± SEM from three independent experiments. The number of cells analyzed per cell line is indicated in parentheses. **b** Frequency distribution of migration speeds from panel A. **c** Live cell migration tracks of the indicated cell lines on fibronectin-coated plates. Each line represents the migration path of a single cell over a 6-h period (parental: *n* = 102; p66-CR (VC): *n* = 109; p66-CR (WT): *n* = 118; p66-CR (S36A): *n* = 98). The starting point of each cell was superimposed onto the origin (0, 0). Tracks were color-coded based on cell speed (black, < 20 μm/h; red, 20–30 μm/h; yellow 30–40 μm/h; green > 40 μm/h). Data represents tracks from three independent experiments. **d** Boyden chamber assays to determine the migratory properties of the indicated cell lines. The data is shown as fold change in cell migration relative to the parental 4T1-537 cell line ± SEM and is representative of 9 wells over three independent experiments. Representative images are shown. For panels **a** and **d**, statistical analysis was performed using a one-way ANOVA with a Tukey’s multiple comparisons test (*P < 0.05; ***P < 0.001; ****P < 0.0001; ^δδδ^*P* < 0.001, and ^δδδ^*P* < 0.0001 represents VC versus p66ShcAWT or VC versus p66ShcAS36A comparisons)

Ruling out an EMT as the primary mechanism by which p66ShcA increased breast cancer lung metastasis, we next examined its impact on the migratory properties of breast cancer cells. Previous research implicated p66ShcA as a mediator of focal adhesion signaling, an essential part of cellular motility [17, 30]. We first assessed whether p66ShcA could promote cell migration by performing live-cell tracking experiments. p66ShcA loss [p66-CR (VC)] reduced the average migration speed (μm/h) of breast cancer cells (2-fold) relative to parental 4T1-537 cells, which could be completely rescued by re-expressing wild-type p66ShcA or the p66ShcA (S36A) mutant (Fig. 4a–c). We confirmed the reduced migratory potential of p66-CR (VC) breast cancer cells, relative to parental 4T1-537 cells, using Boyden chamber assays, which again was rescued both in p66ShcA (WT)- and p66ShcA (S36A)-expressing cells (Fig. 4d). Given that p66ShcA is cytoplasmic, and only a fraction translocates to the mitochondria in response to stress [35], these data suggest that non-mitochondrial p66ShcA pools may increase the migratory properties of mammary tumors, resulting in an increased lung-metastatic potential (Fig. 3b, c). The inability of the p66ShcA (S36A) mutant to metastasize to the lung suggests that this phosphorylation site is essential for different aspects of the metastatic cascade, which occur prior to extravasation into the lung (Fig. 3b, c, e).

**Fig. 5:**
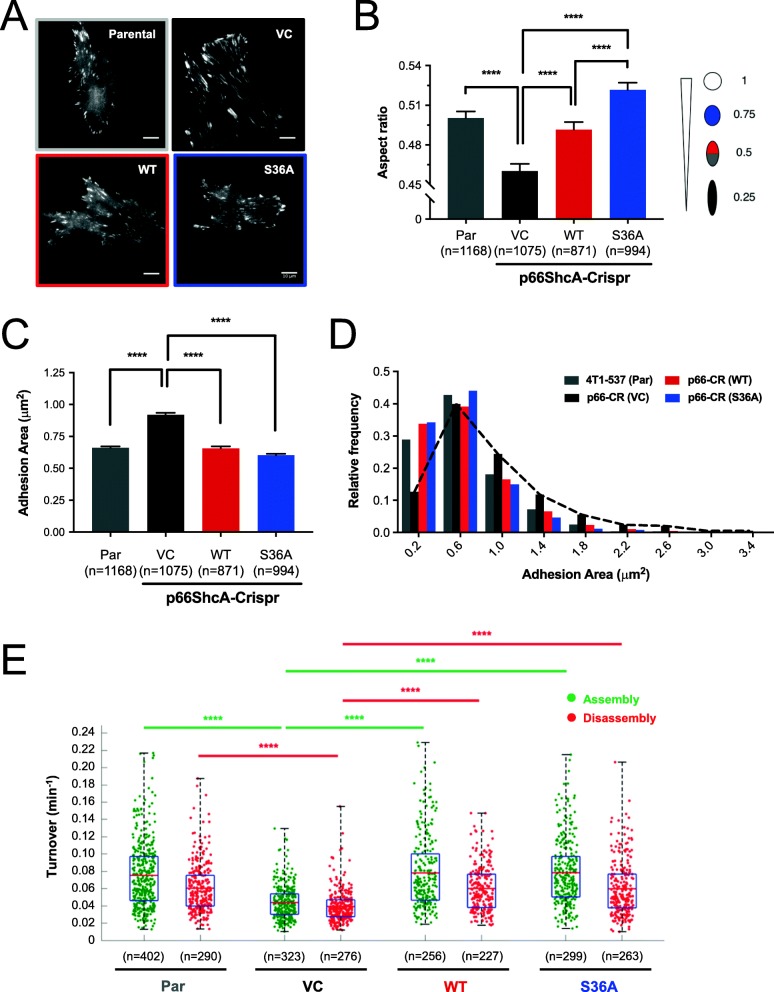
Non-mitochondrial p66ShcA accelerates the dynamics of focal adhesion formation. **a** Representative images of parental, p66-CR (VC), p66-CR (WT), and p66-CR (S36A)-expressing lung-metastatic 4T1 cells (537 population) transfected with mCherry paxillin. Scale bar is 10 μm. **b** Adhesions were segmented based on fluorescence intensity and analyzed for shape. Aspect ratio was determined by finding the ratio between semi-minor and semi-major axes. **c** Adhesions were segmented based on fluorescence intensity and analyzed for size. **d** Frequency distribution of adhesion areas from **c**. Data values were binned into 1-μm^2^ segments. **e** Adhesions in protrusive cell regions were tracked over time to determine average assembly and disassembly rates from changes in mean fluorescence intensity. Cells were imaged every 20 s for a total of 25 min (parental: *n* = 12; VC: *n* = 11; WT: *n* = 10; S36A: *n =* 11). Data represent assembly and disassembly events for adhesions from three independent experiments. Top and bottom lines of the box indicate the 3rd and 1st quartile, respectively, while the bold central lines indicate mean. For panels **b** and **c**, statistical analysis was performed using a one-way ANOVA with a Tukey’s multiple comparisons test (****P < 0.0001). For panel **e**, statistical analysis was performed using a two-way ANOVA with a Tukey’s multiple comparisons test (****P < 0.0001). The whiskers extend up to 1.5 times the interquartile range

Given the importance of wild-type p66ShcA on cellular migration, we further interrogated its role in modulating adhesion dynamics [30]. Focal adhesions are multi-protein complexes that link the actin cytoskeleton of a migrating cell to the extracellular matrix (ECM) through integrin-mediated interactions. Force-dependent assembly and disassembly of focal adhesions dynamically controls cell motility and metastasis. Fibrillar adhesions are larger, more mature, and elongated structures that stabilize cell-matrix interactions to reduce migratory speed. Force-bearing focal adhesions are smaller, yet mature, structures that facilitate the generation of tractional force necessary for cell migration [36]. To study focal adhesion dynamics, breast cancer cells were transfected with a mCherry-paxillin fusion protein, an adhesion marker, and imaged over time using total internal reflection fluorescence (TIRF) microscopy. Representative images were first analyzed to compare focal adhesion shape and size (Fig. 5a). The shape of cellular adhesions was quantified by calculating aspect ratios, representing the degree of circularity for a single adhesion on a scale from 0 to 1 (with 1 being the most circular and 0 representing more elongated shapes) (Fig. 5b). These analyses revealed that p66-CR (VC) cells had the lowest aspect ratios (most elongated adhesions), whereas 4T1-537 parental and p66-CR (WT) cells exhibited higher and similar aspect ratios. Finally, p66-CR (S36A) cells possessed the highest aspect ratio, indicating the greatest degree of circularity (Fig. 5b). We also measured whether p66ShcA altered the size of cellular adhesions. We show that p66-CR (VC) cells contained significantly larger adhesions relative to any other cell population with respect to the average area of cellular adhesions (Fig. 5c) and their relative distribution based on size (Fig. 5d). In contrast, 4T1-537 parental, p66-CR (WT), and p66-CR (S36A) cells formed smaller, comparably sized, adhesions (Fig. 5c, d). Thus, p66ShcA loss promotes the formation of larger, more elongated cellular adhesions, indicative of fibrillar structures that restrain cell migration. Non-mitochondrial p66ShcA pools enable the formation of smaller, more circular cellular adhesions that may increase cell migration. Consistent with their increased size and maturity, adhesions from p66ShcA-deficient breast cancer cells [p66-CR (VC)] had the lowest rates of assembly and disassembly (Fig. 5e). In contrast, 4T1-537 parental, p66-CR (WT), and p66-CR (S36A) cells exhibited comparable adhesion dynamics (Fig. 5e).

### p66ShcA is dispensable for the formation of functional invadopodia and ECM degradation

Finally, we assessed whether p66ShcA altered the proteolytic property of breast cancer cells by plating them onto fluorescently-labeled gelatin and measuring the degree of gelatin degradation. Surprisingly, we found that p66-CR (VC) cells showed the greatest increase in gelatin degradation (Additional file 8: Figure S8A), even though they displayed the lowest metastatic potential (Fig. 3b, c). However, both p66-CR (WT)- and p66-CR (S36A)-expressing cells displayed a similar degree of gelatin degradation relative to parental controls (Additional file 8: Figure S8A). Examination of the degraded surface area in these studies revealed two distinct degradation patterns: (1) “punctate” areas of gelatin degradation suggestive of individual invadopodia and (2) larger “patches” of gelatin degradation that may arise due to sustained invadopodia formation in cells that are less migratory. Indeed, most of the degradation patterns observed with p66-CR (VC) cells (80%) resembled these larger degradation patterns (Additional file 8: Figure S8B), which is consistent with their reduced migratory speed (Fig. 4), larger focal adhesion structures (Fig. 5c, d), and impaired rates of adhesion assembly and disassembly (Fig. 5e). In contrast, 4T1-537 (Par)-, p66-CR (WT)-, and p66-CR (S36A)-expressing cells form a higher frequency of more “punctate” degradation patterns (Additional file 8: Figure S8B), consistent with their increased migratory properties and adhesion dynamics (Figs. 4 and 5). Finally, we show that the degree of gelatin degradation was comparable across all groups when specifically analyzing areas with punctate degradation patterns, resembling invadopodia (Additional file 8: Figure S8C). These data suggest that neither p66ShcA loss [p66-CR (VC)], nor expression of p66ShcA^WT^ or p66ShcA^S36A^ alleles, appreciably altered the degradative properties of breast cancer cells. However, p66-CR (VC) cells degraded a significantly larger surface area of gelatin, compared to p66ShcA^WT^ and p66ShcA^S36A^-expressors, when specifically examining larger patches of degradation (Additional file 8: Figure S8D). These data argue the increased degradative behavior of p66ShcA-null cells may be an indirect effect of their reduced cell migration and increased adhesion to the extracellular matrix, resulting in the formation of larger and more stable degradative structures.

### Distinct p66ShcA pools support breast cancer cell survival in circulation and subsequent lung colonization

**Fig. 6:**
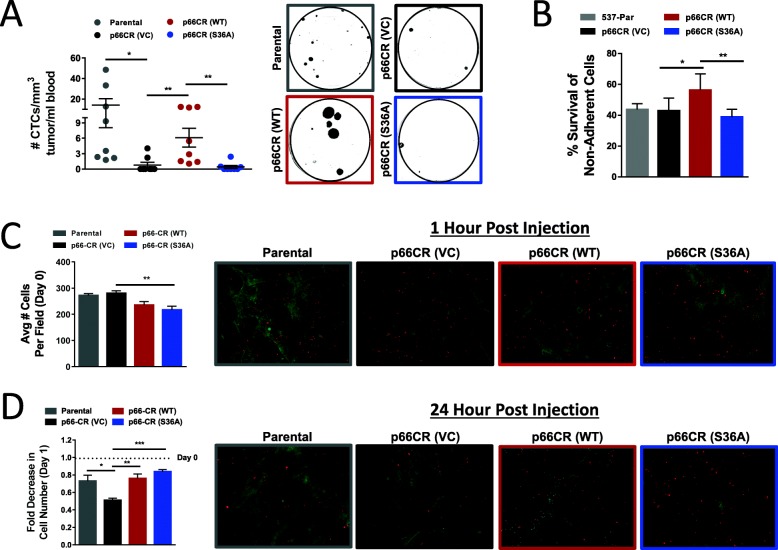
p66ShcA increases breast cancer cell dissemination into the bloodstream and subsequent lung colonization. **a** Number of circulating tumor cells (CTC) in mice bearing parental, p66-CR (VC), p66-CR (WT), and p66-CR (S36A) mammary tumors normalized both to tumor volume at necropsy and the volume of blood collected. The data is shown as average number of CTCs/mm^3^ tumor/ml blood ± SEM and is representative of 8 mice per group. **b** Percentage of surviving cells following matrix detachment (anoikis resistance) was determined for 4T1-537 parental, p66-CR (VC), p66-CR (WT), and p66-CR (S36A) cells. The percentage of surviving cells was normalized to that observed for adherent cells for each cell line. The data is representative of five replicates from three independent experiments. **c** Number of breast tumor cells present in the lung 1 h following tail vein injection. Breast cancer cells were labeled with Cell Tracker Red CMPTX dye and visualized in the lungs by fluorescent microscopy. The data is shown as average # cells per field of view ± SEM. For each cell line, the data is representative of 5 mice and 5 fields of view per mouse. Representative fluorescent images are shown. **d** Breast cancer cells were labeled and visualized as in panel **b**. The data is shown as the fold decrease in cell number 24 h post tail vein injection relative to 1 h ± SEM. The data is representative of 5 mice and 5 fields of view per mouse. Representative fluorescent images are shown. For panels **a**–**d**, statistical analysis was performed using a one-way ANOVA with a Tukey’s multiple comparison test (*P < 0.05; **P < 0.01; ***P < 0.001)

Whereas both p66ShcA-WT- and p66ShcA-S36A-expressing cells increase breast cancer cell migration, p66-CR (S36A) tumors are severely debilitated in their ability to spontaneously metastasize (Fig. 3b, c). However, p66ShcA-S36A-expressing cells could efficiently seed the lungs following tail vein injection (Fig. 3e). These data support an important role for Ser36 phosphorylation of p66ShcA in increasing the ability of breast cancer cells to intravasate into the bloodstream and survive in circulation. To test this, we quantified the number of circulating tumor cells (CTCs) in mice that developed primary mammary tumors following injection of 4T1-537 parental, p66-CR (VC), p66-CR (WT), and p66-CR (S36A) breast cancer cells. These data revealed fewer CTCs in the bloodstream of mice bearing p66ShcA null tumors compared to parental controls (Fig. 6a). Consistent with their ability to spontaneously metastasize, wild-type p66ShcA partially rescued the number of CTCs, whereas the non-phosphorylatable p66ShcA mutant failed to do so (Fig. 6a). In parallel, we measured the ability of these cells to survive following matrix detachment in vitro. We do not observe appreciable differences in the degree of anoikis between 4T1-537 parental and p66ShcA-deficient cells, which is likely reflective of the culture conditions. However, restoration of wild-type p66ShcA into p66-CR (VC) cells increases survival of breast cancer cells following matrix detachment, which is abrogated with the p66ShcA (S36A) mutant (Fig. 6b). The inability of the p66ShcA (S36A) mutant to become phosphorylated and/or translocate into the mitochondria may increase cancer cell anoikis in circulation, leading to the reduced spontaneous lung-metastatic potential observed in p66ShcAS36A-expressing breast tumors from the orthotopic site (Fig. 3c, Additional file 1: Figure S1E). These data suggest that phosphorylation of serine 36 in p66ShcA is important to increase survival of circulating tumor cells.

Following intravasation into the bloodstream, breast tumor cells must successfully extravasate and seed the secondary organ to support their metastatic dissemination. To test this, we enumerated the ability of fluorescently labeled cancer cells to colonize the lung immediately following tail vein injection. The lungs of mice injected with parental, p66-CR (VC), p66-CR (WT), and p66-CR (S36A) breast cancer cells were examined by whole-mount fluorescent microscopy either 1 h or 24 h following tail vein injection. At 1 h post-injection, the number of cancer cells in the lungs of mice from each cohort was similar, although there were slightly fewer cells in the lungs of mice bearing p66ShcA (S36A)-expressing cells (Fig. 6c). By comparing the ratio of fluorescently labeled cells remaining in the lung 24 h post-injection, relative to the 1-h time point, we calculated the ability of breast cancer cells to colonize and survive in the lung immediately following extravasation. Loss of p66ShcA significantly reduced the proportion of surviving cells relative to p66ShcA-proficient controls (Fig. 6d). Moreover, both wild-type p66ShcA and p66ShcA (S36A) alleles rescued lung colonization in p66ShcA-null cells (Fig. 6d). These data suggest that non-mitochondrial p66ShcA pools promote efficient breast cancer cell lung colonization.

### Non-mitochondrial p66ShcA pools increase the growth potential of breast cancer lung metastases

**Fig. 7:**
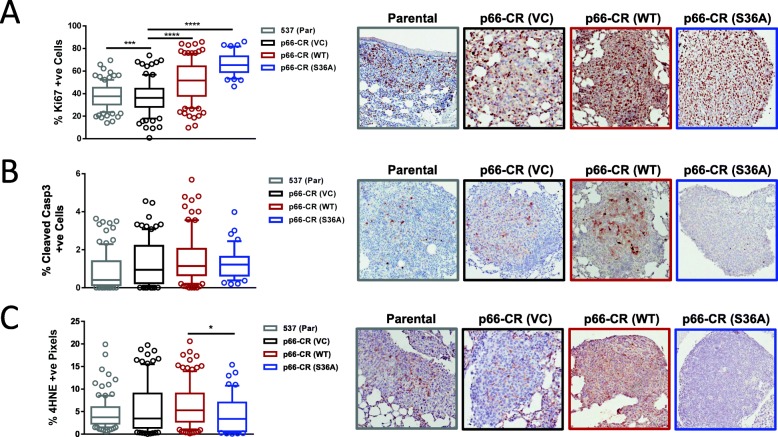
Non-mitochondrial role for p66ShcA in increasing the growth of macroscopic breast tumor lung metastases. **a** Percentage of Ki67-positive cells, **b** cleaved Caspase-3 positive cells, and **c** 4HNE positive pixels in individual lung-metastatic lesions derived from 4T1-537 Parental, p66-CR (VC), p66-CR (WT), and p66-CR (S36A) breast cancer cells, both following tumor resection and following tail vein injection. Panel **a**: Parental, *n* = 109 lesions; p66-CR (VC), *n* = 90 lesions; p66-CR (WT), *n* = 116 lesions; p66-CR (S36A), *n* = 45 lesions. Panel **b**: Parental, *n* = 108 lesions; p66-CR (VC), *n* = 89 lesions; p66-CR (WT), *n* = 89 lesions; p66-CR (S36A), *n* = 45 lesions. Panel **c**: Parental, *n* = 111 lesions; p66-CR (VC), *n* = 95 lesions; p66-CR (WT), *n* = 116 lesions; p66-CR (S36A), *n* = 55 lesions. Representative IHC images are also shown. For panels **a** and **c**, statistical analysis was performed using a one-way ANOVA with a Tukey’s multiple comparisons test (*P < 0.05; ***P < 0.001; ****P < 0.0001)

The final stage of the metastatic cascade requires that disseminated breast cancer cells survive and thrive in an oxidative lung microenvironment to promote the formation of macroscopic metastases. We therefore assessed whether and how p66ShcA impacts proliferation, apoptosis, and oxidative damage in breast cancer lung metastases. This was achieved via Ki67, cleaved Caspase-3, and 4HNE (a marker of ROS-induced lipid peroxidation) IHC staining of macroscopic lung metastases. Whereas p66ShcA is dispensable for breast cancer proliferation in the primary tumor (Additional file 5: Figure S5A), re-constitution of p66ShcA-null breast tumors with either p66ShcA-(WT) or the p66ShcA-(S36A) mutant increased the proliferative rate of macroscopic breast cancer lesions (Fig. 7a). These results suggest that non-mitochondrial p66ShcA pools increase proliferation of 4T1 lung-metastatic breast cancer cells, specifically in the lung microenvironment.

Given that p66ShcA is a redox protein and the lung microenvironment has a high oxidative potential, we also assessed the degree of oxidative damage along with induction of an apoptotic response. We show that wild-type p66ShcA was dispensable for controlling breast cancer cell survival, both within the lung metastases (Fig. 7b) and primary tumors (Additional file 6: Figure S6). Similarly, loss of p66ShcA did not change the degree of lipid peroxidation (4HNE) in either the primary tumor (Additional file 9: Figure S9) or lung metastases (Fig. 7c). Re-expression of the non-phosphorylatable form of p66ShcA (S36A) modestly reduced the degree of lipid peroxidation in breast cancer lung metastases but had no appreciable impact on their apoptotic rate (Fig. 7b, c).

### Increased AKT/mTOR and Src family kinase signaling is associated with the pro-metastatic properties of non-mitochondrial p66ShcA pools

Overall, we demonstrate an essential role for p66ShcA in increasing the lung-metastatic potential of aggressive TNBC cells. Our data using a p66ShcA mutant that cannot translocate to the mitochondria reveal that this adaptor protein potentiates several steps of the metastatic cascade independently of its ability to induce mitochondrial ROS formation. This includes increased cell migration, lung colonization, and growth of secondary metastases. However, our data suggests a unique role for mitochondrial p66ShcA in facilitating the intravasation into and/or survival of circulating tumor cells within the bloodstream. In order to elucidate potential mechanisms that contribute to p66ShcA-induced lung metastasis, we assessed the status of several signaling pathways that have previously been implicated in this process. In order to model stress responses that breast cancer cells are likely to encounter in vivo, we cultured the 4T1-537 cells that differ in their p66ShcA status (p66ShcA-null, p66ShcA-WT, or p66ShcA-S36A) under conditions that alter nutrient availability (10% vs 0% FBS), anchorage dependency (adherent versus suspension), and oxidative stress (phenformin, arsenic).

We first focused on signaling pathways controlling energy balance, given the central requirement for increased bioenergetics in promoting breast cancer lung metastasis [37, 38]. Indeed, p66ShcA has been shown to support catabolic metabolism by favoring a shift towards oxidative phosphorylation [39]. We therefore examined whether modulating p66ShcA function altered AMPK activation, a ser/thr kinase that is a central regulator of metabolic reprogramming towards catabolic reactions [40]. Interestingly, we observed increased AMPK phosphorylation, specifically in p66ShcA-S36A-expressing cells, under nutrient replete conditions (Additional file 10: Figure S10A). However, in response to nutrient deprivation, cell detachment, or oxidative stress conditions, AMPK is activated in a p66ShcA-independent manner within all cell lines (Additional file 10: Figure S10A). Moreover, we do not observe appreciable pAMPK levels in any of the mammary tumors or lung metastases that we analyzed (Additional file 10: Figure S10B). These data suggest that although non-mitochondrial p66ShcA pools may regulate AMPK to control cellular bioenergetics under normal growth conditions, deregulation of this pathway is not likely to contribute to the increased ability of p66ShcA to promote breast cancer lung metastasis.

We next examined whether Src family kinase (SFK) signaling was altered in the various breast cancer cell lines that differ in their lung-metastatic potential, given their multi-faceted role in controlling several stages of the metastatic cascade [41]. We show that p66ShcA-S36A-expressing cells specifically exhibit increased tyrosine phosphorylation of SFKs in vitro (Additional file 11: Figure S11A) and in mammary tumors in vivo (Additional file 11: Figure S11B). However, SFK activation is further potentiated in response to nutrient deprivation or oxidative stress, but in a p66ShcA-independent manner. Moreover, SFK Y416-phosphorylation is severely reduced in all cell lines examined following matrix detachment (Additional file 11: Figure S11A). Finally, we also do not observe significant differences in pSFK levels in any of the lung metastases irrespective of p66ShcA status (Additional file 11: Figure S11B). Taken together, these observations suggest that the elevated SFK activation, increased cell migration and focal adhesion dynamics mediated by non-mitochondrial p66ShcA pools (Figs. 4 and 5) may contribute to the ability of p66ShcA to potentiate the early stages of the metastatic cascade (Fig. 3).

**Fig. 8:**
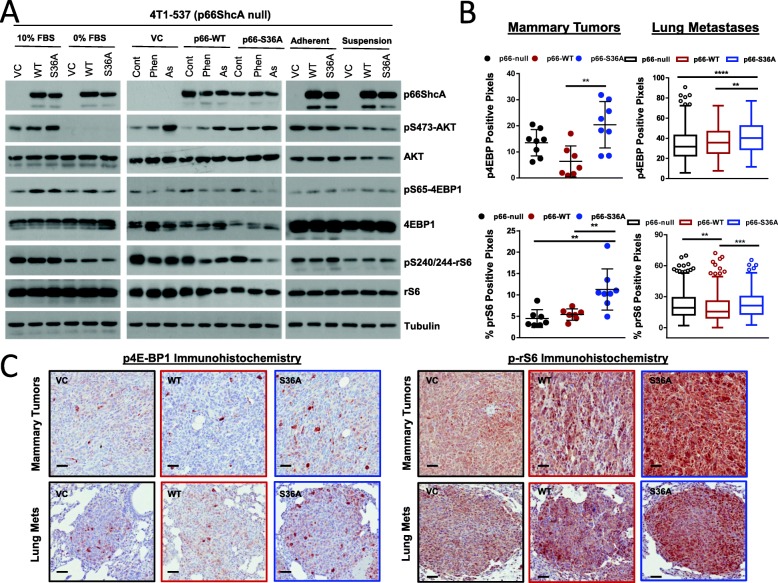
Non-mitochondrial p66ShcA pools activate the AKT/mTOR pathway in breast tumors. **a** Whole-cell lysates were generated from the indicated cell lines grown under the following conditions: 10% FBS versus 0% FBS for 16 h; 1 mM phenformin for 2 h; 20 μM sodium arsenate for 4 h; suspension cultures on ultra-low attachment plates for 16 h. Immunoblot analysis was performed using the indicated antibodies. **b** Percentage of p37/46-4EBP1 and pS240/244-rS6 positive pixels in primary breast tumors (*n* = 8 tumors/genotype) and in individual lung-metastatic lesions derived from 4T1-537 p66-CR (VC), p66-CR (WT), and p66-CR (S36A) breast cancer cells. For the lung metastases: p66-CR (VC), *n* = 235 lesions (p4EBP) and *n* = 306 lesions (p-rS6); p66-CR (WT), *n* = 202 lesions (p4EBP) and *n* = 315 lesions (p-rS6); p66-CR (S36A), *n* = 237 lesions (p4EBP) and *n* = 305 lesions (p-rS6). Statistical analysis was performed using a one-way ANOVA with a Tukey’s multiple comparisons test (**P < 0.01; ***P < 0.001; ****P < 0.0001). **c** Representative IHC images for the data shown in panel **b** are shown

Finally, we studied whether differences in RAS/MAPK or AKT/mTOR signaling correlate with the increased metastatic potential of p66ShcA-expressing breast cancers. Indeed, these pathways are central to breast cancer metastasis [42–44]. Paradoxically, p66ShcA has been shown to suppress mitogenic responses by inhibiting these signaling pathways in non-transformed cells and under conditions of severe oxidative stress [6, 45–47]. However, the role of p66ShcA in controlling Ras/MAPK and AKT/mTOR signaling in cancer cells under chronic, yet moderately elevated stress conditions, remains undefined. Indeed, we do not observe any differences in activation of MAPK family members that control mitogenic (ERK) or stress (p38MAPK, JNK) signaling in p66ShcA-null, p66ShcA-WT, and p66ShcA-S36A-expressing cells under basal conditions. Moreover, whereas biological stressors, including nutrient deprivation, oxidative stress, or ECM detachment, increased p38MAPK and/or JNK activation, they did so in a p66ShcA-independent manner (Additional file 12: Figure S12). Our data argue that MAPK family members are unlikely to be central for the observed phenotypes; thus, we next focused on the AKT/mTOR pathway. We show that p66ShcA-S36A-expressing cells exhibit increased AKT activation compared to those lacking p66ShcA or expressing p66ShcA-WT, specifically under pro-mitogenic conditions (Fig. 8a). This is coincident with increased phosphorylation of downstream mTOR targets both in p66ShcA-S36A-expressing cells in vitro (p4EBP1) (Fig. 8a) and mammary tumors in vivo (p4EBP1 and prS6) (Fig. 8b). However, we observe more modest effects on 4E-BP and rS6 phosphorylation in lung metastases (Fig. 8b). Taken together, these data suggest that non-mitochondrial p66ShcA pools may augment breast cancer metastasis by engaging the AKT/mTOR pathway to potentiate numerous stages of the metastatic cascade.

## Discussion

Breast cancers are lethal primarily due to metastatic progression. We now show that the p66ShcA adaptor protein, which is variably expressed in breast cancers, enhances the metastatic potential of breast cancers. Whereas p66ShcA overexpression is not sufficient to increase breast cancer lung metastasis in two independent models, we show that breast cancer cells with increased lung-metastatic potential upregulate endogenous p66ShcA levels and that p66ShcA is necessary for these cells to retain their lung-metastatic fitness. These data support the idea that p66ShcA is one of many pro-metastatic genes that may be coordinately upregulated and collaboratively increase the lung-metastatic potential of breast cancers. Identification of such coordinately regulated genes is beyond the scope of this study but warrant further investigation.

**Fig. 9:**
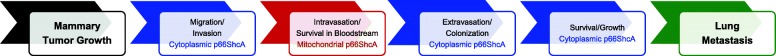
Distinct intracellular p66ShcA pools collaborate to potentiate breast cancer lung metastasis. Schematic diagram illustrating how both cytoplasmic and mitochondrial p66ShcA pools collaborate to potentiate discrete stages of the metastatic cascade. Altogether, breast cancer cells rely on both mitochondrial and cytoplasmic p66ShcA complexes to increase their lung-metastatic potential

We further show that both mitochondrial and cytoplasmic p66ShcA pools collaborate to ensure the metastatic fitness of breast cancer cells (Fig. 9). Indeed, we identified a unique role for retention of the Ser36 phosphorylation site of p66ShcA, which controls its mitochondrial localization, for efficient survival of intravasating circulating tumor cells into the bloodstream. This observation is consistent with studies indicating that mitochondrial ROS is required for efficient breast cancer lung metastasis from the primary site [48, 49]. Our data with a non-phosphorylatable p66ShcA-(S36A) mutant further suggests that this adaptor protein also promotes other key steps in the metastatic cascade, including migration, focal adhesion turnover, lung colonization, and outgrowth of secondary metastases, independently of its ability to stimulate mitochondrial ROS production (Fig. 9). This is consistent with previous studies demonstrating that reactive oxygen species also inhibit the lung-metastatic potential of cancer cells following colonization, necessitating that they acquire adaptive responses to combat oxidative stress in the lung microenvironment [50, 51]. Furthermore, we do not observe evidence for increased oxidative stress in breast cancer lung metastases. These observations indicate that mitochondrial and non-mitochondrial p66ShcA pools collaborate to control distinct steps of the metastatic cascade.

Even though p66ShcA induces an EMT in NIC tumors, which are models for luminal breast cancer [23], our data suggest that a p66ShcA-induced EMT is not sufficient to further promote lung metastasis of NIC tumors. Moreover, we do not observe differences in expression of epithelial or mesenchymal markers in 4T1 tumors, which is a model for triple-negative breast cancer. Despite this fact, p66ShcA is required to support the lung-metastatic potential of 4T1 tumors. These data are consistent with independent studies demonstrating that an EMT may be dispensable for the ability of pancreatic and breast cancer cells to metastasize to the lung [52, 53]. Nevertheless, these observations do not rule out the possibility that breast cancer cells undergoing an EMT can be pro-metastatic. For example, following an EMT, cancer cells can act in paracrine to increase the metastatic properties of neighboring cells [54]. Our data suggests that a causal relationship between EMT and breast cancer lung metastasis is likely context dependent.

To define the mechanisms by which p66ShcA enhances metastasis, we therefore decided to examine the role of p66ShcA in breast cancer cell motility, partly guided by previous research showing that it can localize to focal adhesions and mediate signaling [14, 16, 17]. Our results demonstrate that p66ShcA loss severely reduces breast cancer motility in vitro due to the formation of larger and more stable cellular adhesions. It is well documented that preventing FAK-mediated adhesion turnover results in larger adhesions with reduced cell speed [55, 56]. Recent research also demonstrates that newly forming adhesions have smaller areas and more circular shapes [57]. These data suggest that the reduced speed of p66ShcA-deficient breast cancer cells is a consequence of mature adhesion formation, which impairs cell migration. Indeed, the assembly and disassembly rates of adhesions in p66ShcA-deficient breast cancer are significantly slower than cells expressing wild-type p66ShcA. Furthermore, p66ShcA (S36A)-expressing cells migrate even faster than their wild-type counterparts, which is consistent with increased Src activation in these cells. Non-mitochondrial p66ShcA pools therefore function to increase cell motility by enhancing adhesion dynamics, potentially through the ability of Src family kinases to modulate actin dynamics. This conclusion is supported by evidence that p66ShcA mediates FAK signaling through RhoA [16, 17].

We also found that non-mitochondrial p66ShcA improved the lung colonization ability of breast cancer cells and subsequently increased their proliferative potential to form overt metastases. One possibility is that p66ShcA may also be mediating integrin signaling at the metastatic site, as it has been found that p66ShcA can facilitate proliferative signaling in adherent cells through RhoA and the YAP/TAZ transcription factors [17]. p66ShcA may also regulate signaling through other interactions. For example, p66ShcA has been reported to activate Rac1 [25], which has important roles in promoting breast cancer cell motility, invasion, and survival [58].

Not all pro-metastatic functions of p66ShcA can be attributed to the non-mitochondrial pool of p66ShcA. Tumors expressing a p66ShcA mutant that cannot translocate to the mitochondria [7] revealed a significantly reduced lung-metastatic burden concomitant with a decreased number of circulating tumor cells and an impaired ability to survive following ECM detachment. The involvement of p66ShcA in anoikis has been well documented for lung cancers, in which p66ShcA is often repressed or silenced [13, 14]. Previous studies have shown that upon recruitment to cellular adhesions, p66ShcA/FAK complexes promote RhoA activation by recruiting Rho-specific GEFs, including GEF-H1 and p115-RhoGEF [17]. In turn, increased RhoA activity initiates anoikis following matrix detachment [16]. Whereas serine 36 phosphorylation of p66ShcA is essential for mitochondrial ROS production, opening of the permeability transition pore and apoptosis [7, 10], paradoxically, p66ShcA serine 36 phosphorylation is protective against anoikis [14, 16]. Indeed, a phospho-mimetic p66ShcA (S36E) mutant was previously shown to promote anoikis resistance in lung cancer cells, thereby promoting their ability to colonize and grow in the lung parenchyma [14]. This is consistent with our observations, which reveal that loss of p66ShcA serine 36 phosphorylation in metastatic breast cancer cells abrogates their ability to survive in circulation and sensitizes them to anoikis. While serine 36 phosphorylation of p66ShcA is likely to be protective of anoikis upon matrix detachment, the precise mechanisms controlling this phenotype remain poorly defined and warrant further investigation.

The dichotomous roles of p66ShcA indicate that this protein has a range of functions. While ShcA acts as a metastasis suppressor in lung cancer, it is a metastasis promoter in breast cancer. The impact of such functions on cancer cell phenotypes is likely to result from a combination of internal and external factors that cooperatively influence which role p66ShcA plays in cancer progression. Future research to better understand how distinct interacting molecules govern the function of p66ShcA, in a context-dependent manner, may lead to the identification of more suitable prognostic indicators or novel therapeutic targets for breast cancer metastases.

## Conclusions

We identify the p66ShcA redox protein as novel mediator of breast cancer lung metastasis. We show that p66ShcA is not sufficient to improve the metastatic capabilities of models representing triple-negative or luminal breast cancer. However, breast cancer cells that have already acquired increased lung- and liver-metastatic potential upregulated endogenous p66ShcA levels and require p66ShcA to promote efficient lung metastasis. These data suggest that p66ShcA acts collectively with a group of pro-metastatic genes, representing cooperative adaptations selected for in breast cancer cells with an increased propensity to metastasize. Moreover, p66ShcA re-expression increased breast cancer metastasis both in spontaneous and experimental metastasis assays, suggesting that p66ShcA acts upon multiple stages of the metastatic cascade. Interestingly, however, tumors expressing a p66ShcA mutant that cannot localize to the mitochondria had reduced spontaneous metastatic burden but retained the ability to seed and colonize the lung following intravasation.

## Supplementary information

**Additional file 1: Figure S1.** Non-mitochondrial p66ShcA restrains metastatic progression in a luminal breast cancer model. **(A)** Immunoblot analysis of vector control (VC), p66ShcA-WT and p66ShcA-S36A overexpressing NIC cells using ShcA- and Tubulin-specific antibodies. **(B)** Mammary fat pad (MFP) injection of VC, p66ShcA-WT and p66ShcA-S36A overexpressing NIC cells. The data is shown as average tumor volume (mm^3^) ± SEM (*n* = 7 tumors/group). Immunohistochemical staining of the indicated mammary tumors using **(C)** Ki67 and **(D)** cleaved Caspase-3 specific antibodies. Representative images are shown. **(E)** Percentage of tumor burden in the lungs of mice bearing VC, p66ShcA-WT and p66ShcA-S36A overexpressing NIC tumors. Mammary tumors were resected at 500 mm^3^ and the development of lung metastases was quantified 28 days later. The data is shown as average lung tumor burden ±SEM (*n* = 9–12 mice/group). Representative images are shown. Statistical analysis was performed using a one-way Anova with a Tukey’s multiple comparisons test (*P < 0.05; **P < 0.01; ***P < 0.001; ****P < 0.0001).

**Additional file 2: Figure S2.** p66ShcA is overexpressed in lung and liver metastatic triple negative breast cancer cells. **(A)** Whole cell lysates were generated from parental 4T1 cells along with explants isolated either from breast tumors (BT: 148,152) or that are enriched for their ability to metastasize to lung (526, 537), liver (2776, 2792) or bone (590, 592). Immunoblot analysis using ShcA- and Tubulin-specific antibodies. **(B)** Individual clones from 4T1 parental tumors were analyzed by immunoblot using a ShcA-specific antibody. **(C)** p66ShcA mRNA levels (transcripts per million) in RNAseq TCGA datasets that include primary breast tumors and metastases. This includes 7 metastases within the luminal A/B and basal subtypes along with 767 primary tumors (also luminal A/B and basal). **(D)** p66ShcA was deleted from 4T1-537 cells by Crispr/Cas9 genomic editing. Individual clones were screened by immunblot analysis using ShcA- and Tubulin-specific antibodies. p66ShcA-null clones identified in red font were pooled to generate p66-CR cells used for further analysis. **(E)** 4T1-537, p66-CR cells were transfected with either empty vector (VC) or p66ShcA-WT or p66ShcA-S36A expression vectors. Immunoblot analysis using ShcA- and Tubulin-specific antibodies.

**Additional file 3: Figure S3.** p66ShcA is phosphorylated on Ser36 in 4T1-537 breast cancer cells. **(A)** Immunoblot analysis of whole cell lysates isolated from 4T1-537 p66-CR (VC), p66-CR (WT) and p66-CR (S36A) breast cancer cells treated with PBS, 1 mM phenformin (2 h) or 20 μM sodium arsenite (4 h) using pSer-p66ShcA, ShcA- and Tubulin-specific antibodies. **(B)** DCFDA flow cytometric staining demonstrating ROS production in response to phenformin or sodium arsenite treatment.

**Additional file 4: Figure S4.** p66ShcA-WT-BirA, but not p66ShcA^S36A^-BirA, translocates to the mitochondria following Actinomycin D treatment. Representative images of streptavidin and Tom20 immunofluorescence in p66-WT-BirA and p66-S36A-BirA expressing 537-4T1 breast cancer cells treated with DMSO (unstimulated) or Actinomycin D. Scale bar is 5 μm.

**Additional file 5: Figure S5.** p66ShcA does not alter the mesenchymal properties of 4T1-derived triple negative breast cancers. **(A)** Immunoblot analysis of whole cell lysates isolated from 4T1-537 parental, p66-CR (VC), p66-CR (WT) and p66-CR (S36A) mammary tumors (*n* = 18 each) using ShcA-, E-Cadherin, Vimentin and Tubulin-specific antibodies. **(B-D)** Densitometric quantification of mammary tumors shown in panel A for the **(B)** p66ShcA/Tubulin, **(C)** p66ShcA/p52ShcA, **(D)** E-Cadherin/Tubulin and **(E)** Vimentin/Tubulin ratios. The data is normalized to the parental 4T1-537 tumors.

**Additional file 6: Figure S6.** p66ShcA minimally impacts the growth properties of lung-metastatic triple negative primary breast tumors. Quantification of the percentage **(A)** Ki67 positive cells and **(B)** cleaved Caspase-3 positive cells in 4T1-537 parental, p66-CR, p66-CR (WT) and p66-CR (S36A) mammary tumors. The data is shown as positivity ± SEM and is representative of 9–10 tumors per group. Representative images are shown below each graph.

**Additional file 7: Figure S7.** A p66ShcA-induced epithelial to mesenchymal transition is dispensable for metastatic progression in a luminal breast cancer model. Immunohistochemical analysis of vector control (VC), p66ShcA-WT and p66ShcA-S36A overexpressing NIC mammary tumors using **(A)** E-Cadherin and **(B)** Vimentin-specific antibodies. The data is shown as average tumor volume (mm^3^) ± SEM (*n* = 7 tumors/group). Representative images are shown. Statistical analysis was performed using a one-way Anova with a Tukey’s multiple comparisons test (*P < 0.05; **P < 0.01; ***P < 0.001; ****P < 0.0001).

**Additional file 8: Figure S8.** ShcA p66 is dispensable for invadopodia formation and gelatin degradation. 488 fluorescently-labeled degradation is represented by signal void. **(A)** Total surface area degraded (μm^2^) was determined from images taken from three independent experiments (*n* = 120 FOV, *n* = 30 each for 4T1-537 (par), p66CR (VC), p66CR (WT) and p66CR (S36A)) and error bars represent s.e.m. **(B)** Punctate and Large gelatin degradation patches, representative images shown, were classified qualitatively. Frequency of degradation pattern taken from quantified images used for (A). **(C)** and **(D)**, group the quantified total surface area degraded (μm^2^) per FOV, from (A), into Punctate and Large degradation patterns, respectively. **(E)** Representative images of quantified gelatin degradation: F-Actin (green) stained by 647-phalloidin and 488-labelled gelatin (grey). Scale bar is 20 μm in length. Statistical analysis performed using a one-way Anova with a Tukey’s multiple comparisons test (*P < 0.05; **P < 0.01; ****P < 0.0001).

**Additional file 9: Figure S9.** p66ShcA does not induce oxidative stress in primary tumors in a model of triple negative breast cancer. Quantification of the percentage of 4-HNE positive pixels in 4T1-537 parental, p66-CR, p66-CR (WT) and p66-CR (S36A) mammary tumors. The data is shown as positivity ± SEM and is representative of 9–10 tumors per group. Representative images are shown below each graph.

**Additional file 10: Figure S10.** AMPK activation is not appreciably regulated by p66ShcA in mammary tumors. (**A**) Whole cell lysates were generated from the indicated cell lines grown under the following conditions: 10% FBS versus 0% FBS for 16 h; 1 mM phenformin for 2 h; 20 μM sodium arsenate for 4 h; suspension cultures on ultra-low attachment plates for 16 h. Immunoblot analysis was performed using pAMPK and AMPK specific antibodies. Percentage of pAMPK positive pixels in primary breast tumors (n = 7–8 tumors/genotype) (**B**) and in individual lung-metastatic lesions (**c**) derived from 4T1-537 p66-CR (VC), p66-CR (WT) and p66-CR (S36A) breast cancer cells. For the lung metastases: p66-CR (VC), *n* = 214 lesions; p66-CR (WT), *n* = 194 lesions; p66-CR (S36A), *n* = 202 lesions. (**B, C**) Representative IHC images for primary breast tumors and lung metastases are shown.

**Additional file 11: Figure S11.** Src family kinase activation is increased by non-mitochondrial p66ShcA pools in mammary tumors. **(A)** Whole cell lysates were generated from the indicated cell lines grown under the following conditions: 10% FBS versus 0% FBS for 16 h; 1 mM phenformin for 2 h; 20 μM sodium arsenate for 4 h; suspension cultures on ultra-low attachment plates for 16 h. Immunoblot analysis was performed using pSFK and Src specific antibodies. Percentage of pSFK positive pixels in primary breast tumors (*n* = 6–7 tumors/genotype) **(B)** and in individual lung-metastatic lesions **(C)** derived from 4T1-537 p66-CR (VC), p66-CR (WT) and p66-CR (S36A) breast cancer cells. For the lung metastases: p66-CR (VC), *n* = 318 lesions; p66-CR (WT), *n* = 308 lesions; p66-CR (S36A), n = 318 lesions. **(B, C)** Representative IHC images for primary breast tumors and lung metastases are shown. Statistical analysis performed using a one-way Anova with a Tukey’s multiple comparisons test (*P < 0.05).

**Additional file 12: Figure S12.** MAPK family members are not regulated by p66ShcA in lung-metastatic 4T1 breast cancer cells. Whole cell lysates were generated from the indicated cell lines grown under the following conditions: 10% FBS versus 0% FBS for 16 h; 1 mM phenformin for 2 h; 20 μM sodium arsenate for 4 h; suspension cultures on ultra-low attachment plates for 16 h. Immunoblot analysis was performed using phospho-specific and total antibodies against ERK, p38MAPK and JNK.

**Additional file 13.** Supplemental methods.

## Data Availability

Not applicable.
