# Supplementary figures and images for: p66ShcA functions as a contextual promoter of breast cancer metastasis
Source: Breast Cancer Res. 2020 Jan 15;22:7. doi: 10.1186/s13058-020-1245-6 (PMC6964019; doi:10.1186/s13058-020-1245-6)

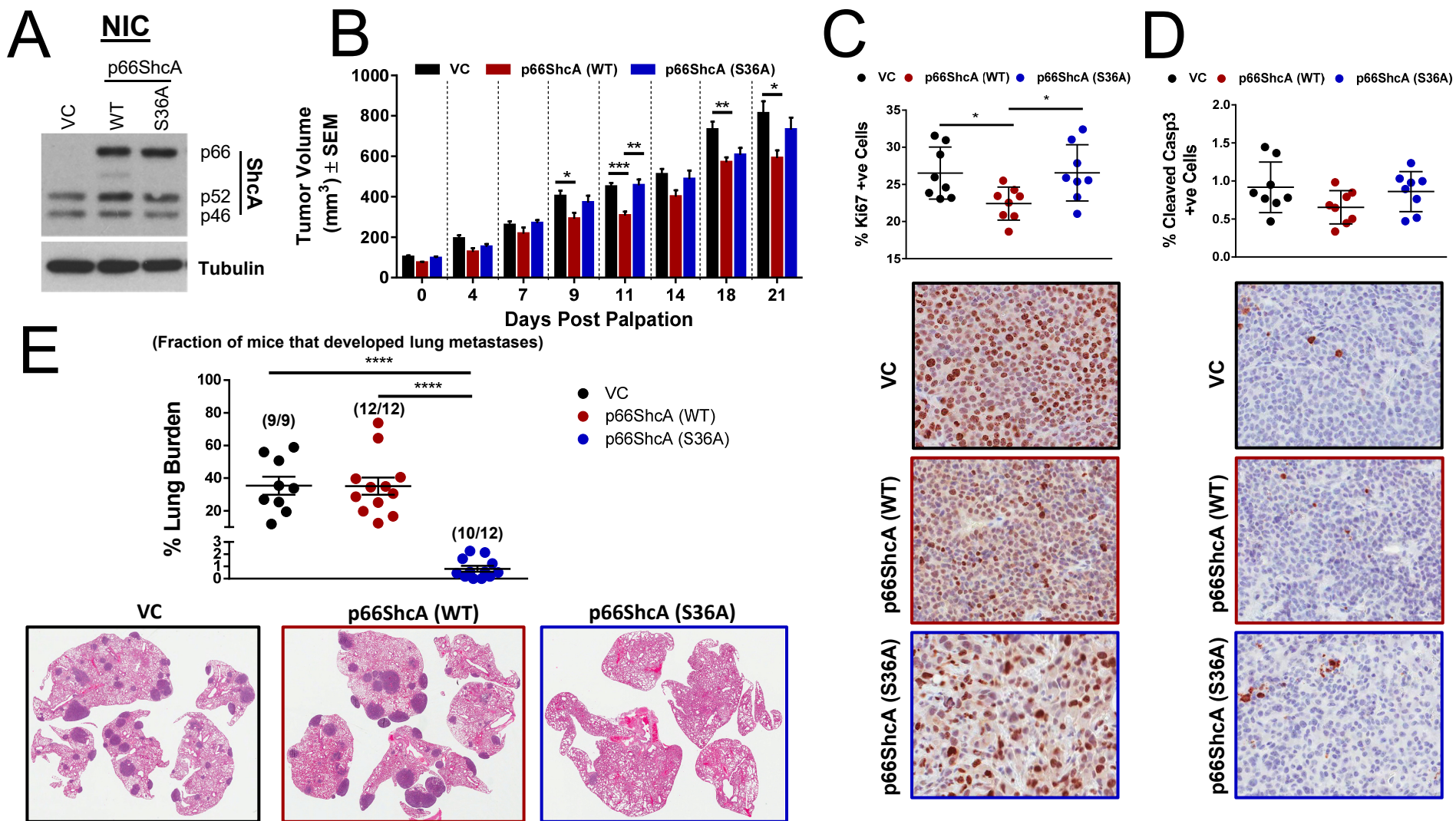

Figure S1

Supplement: Supplementary file 1 — Additional file 1: Figure S1. Non-mitochondrial p66ShcA restrains metastatic progression in a luminal breast cancer model. (A) Immunoblot analysis of vector control (VC), p66ShcA-WT and p66ShcA-S36A overexpressing NIC cells using ShcA- and Tubulin-specific antibodies. (B) Mammary fat pad (MFP) injection of VC, p66ShcA-WT and p66ShcA-S36A overexpressing NIC cells. The data is shown as average tumor volume (mm3) ± SEM (n = 7 tumors/group). Immunohistochemical staining of the indicated mammary tumors using (C) Ki67 and (D) cleaved Caspase-3 specific antibodies. Representative images are shown. (E) Percentage of tumor burden in the lungs of mice bearing VC, p66ShcA-WT and p66ShcA-S36A overexpressing NIC tumors. Mammary tumors were resected at 500 mm3 and the development of lung metastases was quantified 28 days later. The data is shown as average lung tumor burden ±SEM (n = 9–12 mice/group). Representative images are shown. Statistical analysis was performed using a one-way Anova with a Tukey’s multiple comparisons test (*P < 0.05; **P < 0.01; ***P < 0.001; ****P < 0.0001). [file 13058_2020_1245_MOESM1_ESM.pdf]

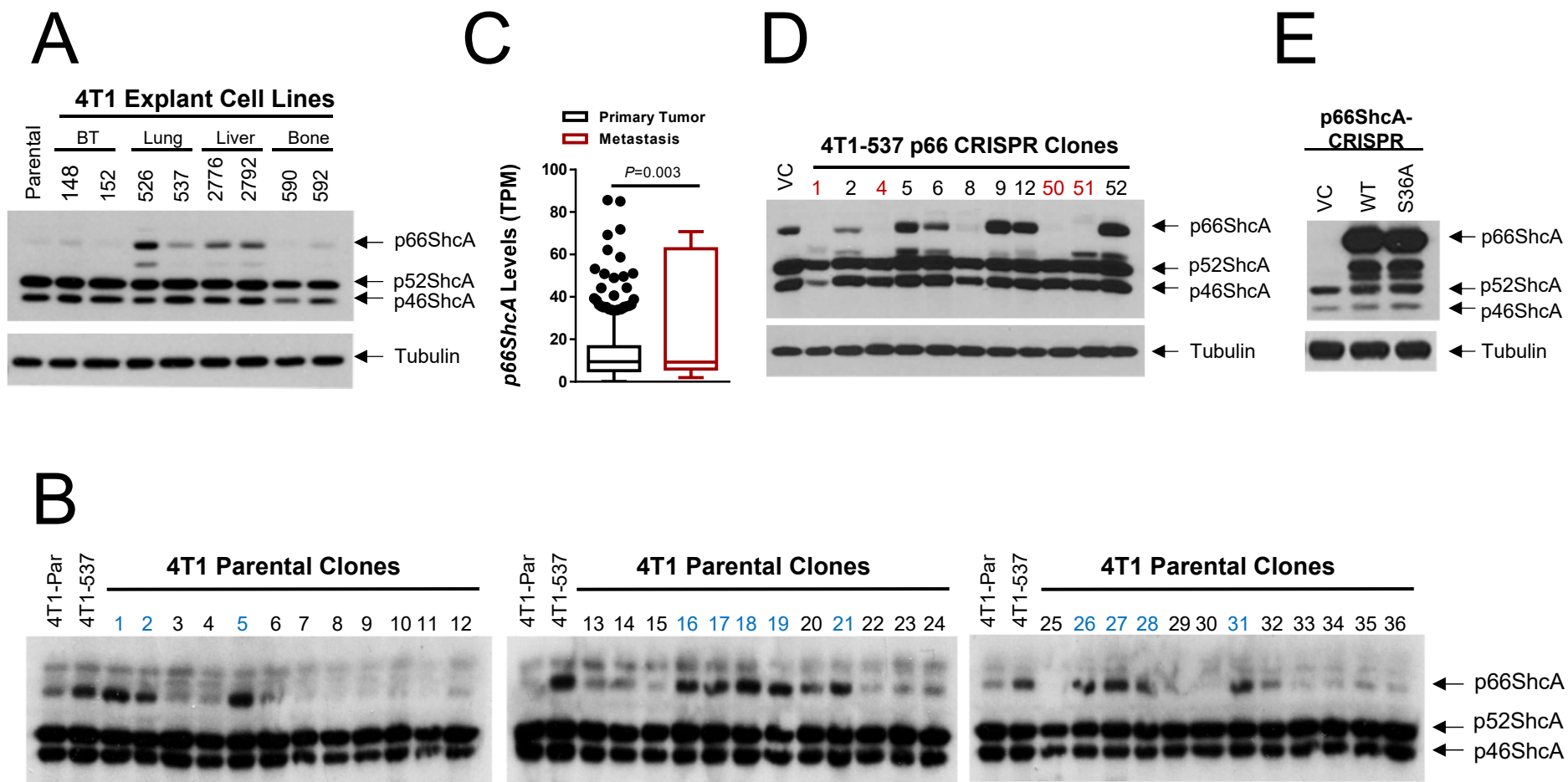

Figure S2

Supplement: Supplementary file 2 — Additional file 2: Figure S2. p66ShcA is overexpressed in lung and liver metastatic triple negative breast cancer cells. (A) Whole cell lysates were generated from parental 4T1 cells along with explants isolated either from breast tumors (BT: 148,152) or that are enriched for their ability to metastasize to lung (526, 537), liver (2776, 2792) or bone (590, 592). Immunoblot analysis using ShcA- and Tubulin-specific antibodies. (B) Individual clones from 4T1 parental tumors were analyzed by immunoblot using a ShcA-specific antibody. (C) p66ShcA mRNA levels (transcripts per million) in RNAseq TCGA datasets that include primary breast tumors and metastases. This includes 7 metastases within the luminal A/B and basal subtypes along with 767 primary tumors (also luminal A/B and basal). (D) p66ShcA was deleted from 4T1-537 cells by Crispr/Cas9 genomic editing. Individual clones were screened by immunblot analysis using ShcA- and Tubulin-specific antibodies. p66ShcA-null clones identified in red font were pooled to generate p66-CR cells used for further analysis. (E) 4T1-537, p66-CR cells were transfected with either empty vector (VC) or p66ShcA-WT or p66ShcA-S36A expression vectors. Immunoblot analysis using ShcA- and Tubulin-specific antibodies. [file 13058_2020_1245_MOESM2_ESM.pdf]

# A

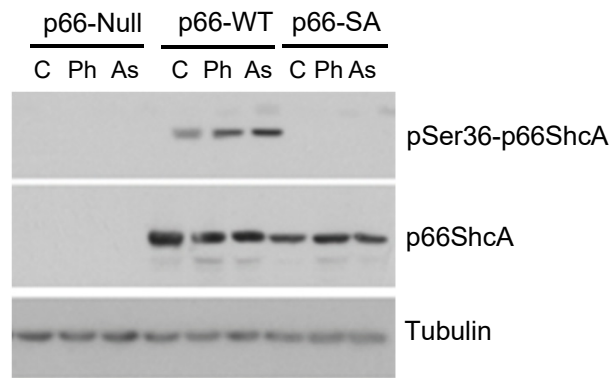

# B

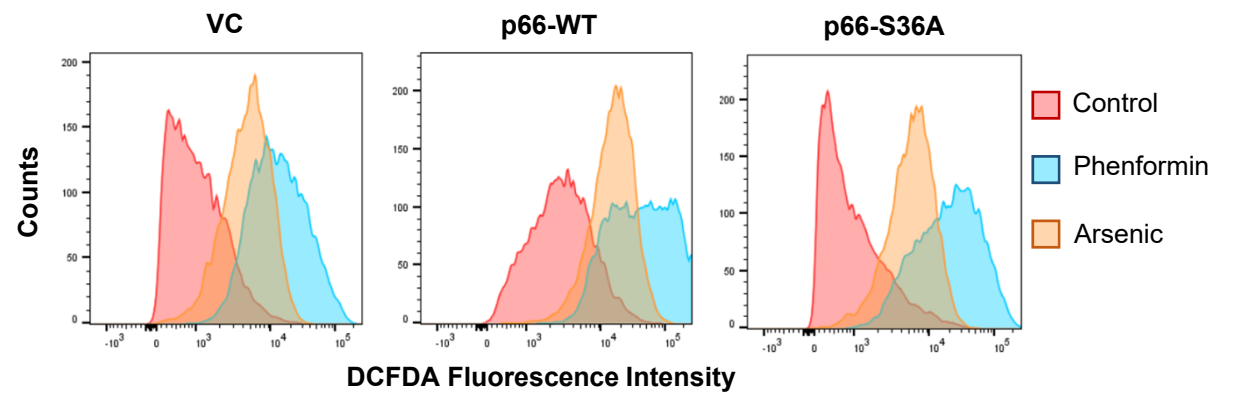

Figure S3

Supplement: Supplementary file 3 — Additional file 3: Figure S3. p66ShcA is phosphorylated on Ser36 in 4T1-537 breast cancer cells. (A) Immunoblot analysis of whole cell lysates isolated from 4T1-537 p66-CR (VC), p66-CR (WT) and p66-CR (S36A) breast cancer cells treated with PBS, 1 mM phenformin (2 h) or 20 μM sodium arsenite (4 h) using pSer-p66ShcA, ShcA- and Tubulin-specific antibodies. (B) DCFDA flow cytometric staining demonstrating ROS production in response to phenformin or sodium arsenite treatment. [file 13058_2020_1245_MOESM3_ESM.pdf]

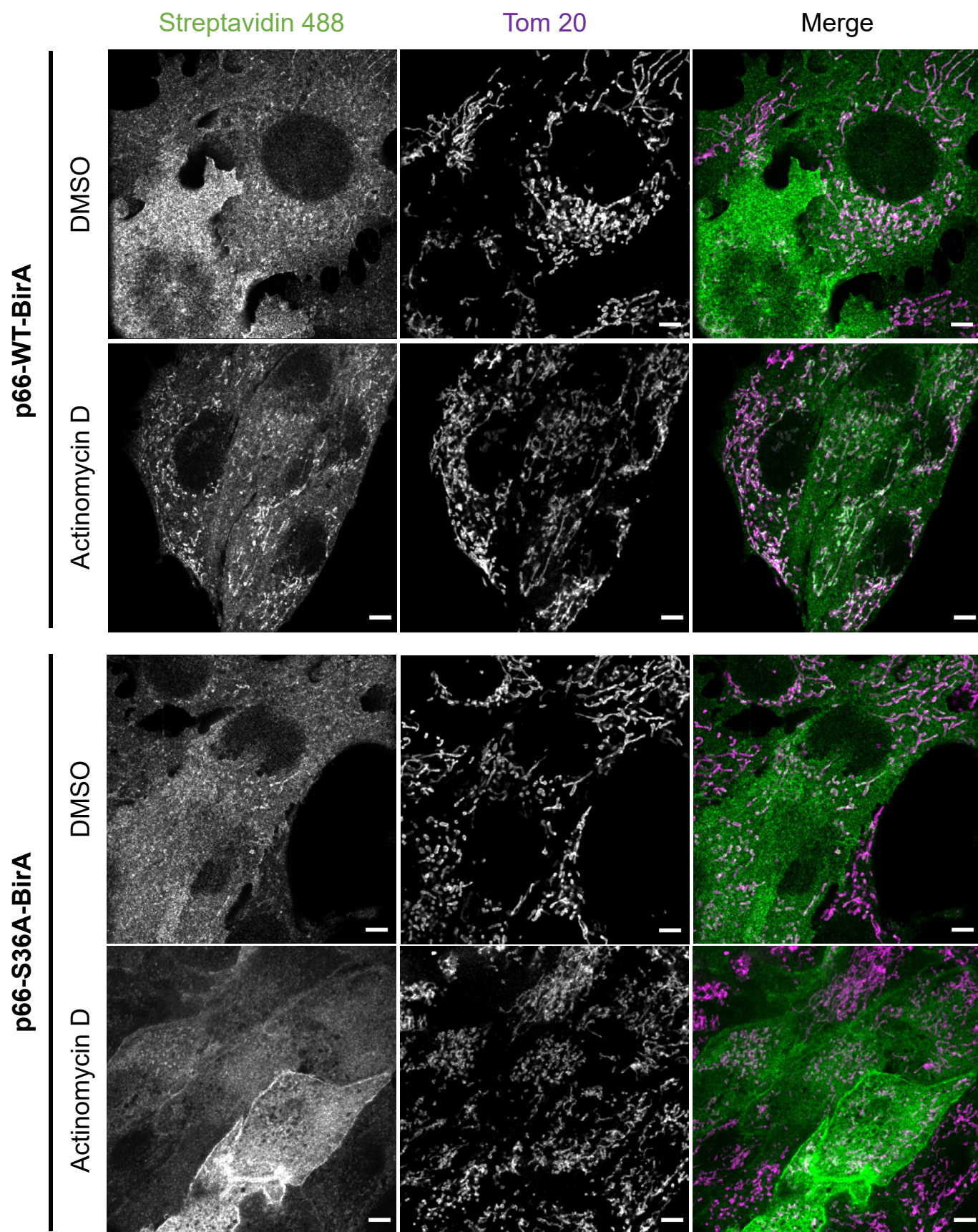

Figure S4

Supplement: Supplementary file 4 — Additional file 4: Figure S4. p66ShcA-WT-BirA, but not p66ShcAS36A-BirA, translocates to the mitochondria following Actinomycin D treatment. Representative images of streptavidin and Tom20 immunofluorescence in p66-WT-BirA and p66-S36A-BirA expressing 537-4T1 breast cancer cells treated with DMSO (unstimulated) or Actinomycin D. Scale bar is 5 μm. [file 13058_2020_1245_MOESM4_ESM.pdf]

Figure S5

A

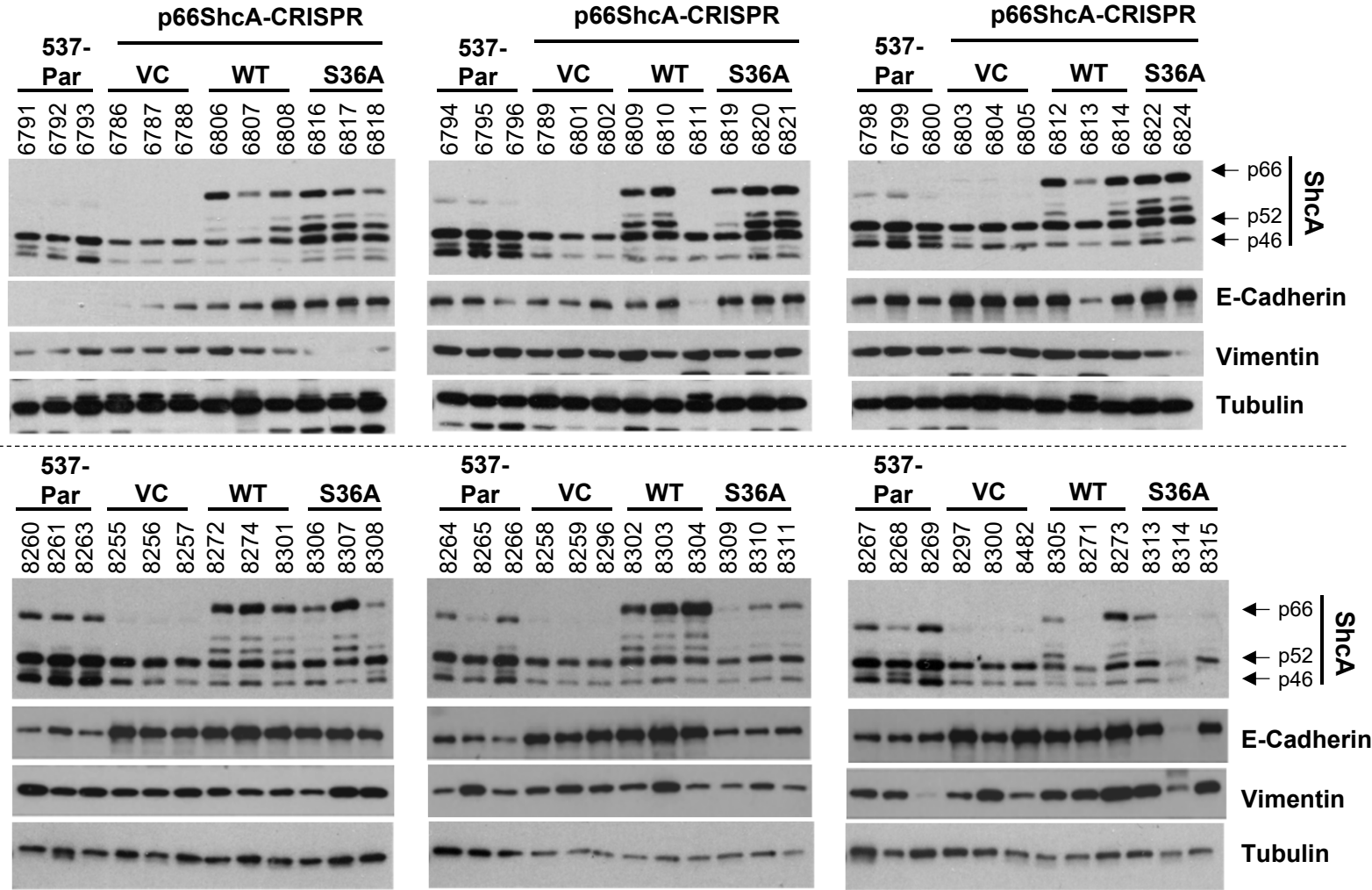

B

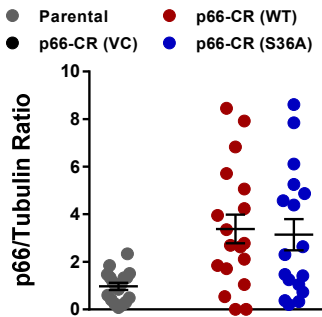

C

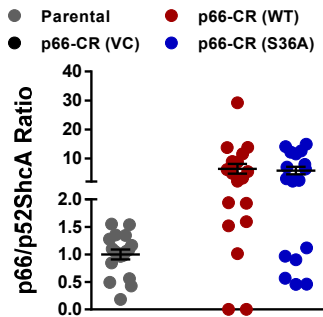

D

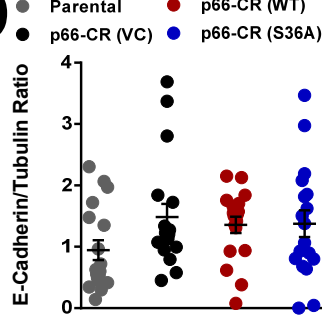

E

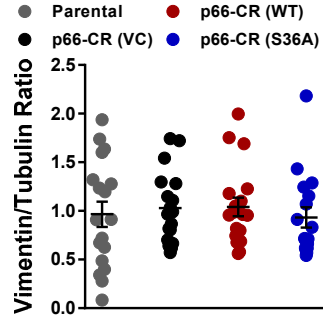

Supplement: Supplementary file 5 — Additional file 5: Figure S5. p66ShcA does not alter the mesenchymal properties of 4T1-derived triple negative breast cancers. (A) Immunoblot analysis of whole cell lysates isolated from 4T1-537 parental, p66-CR (VC), p66-CR (WT) and p66-CR (S36A) mammary tumors (n = 18 each) using ShcA-, E-Cadherin, Vimentin and Tubulin-specific antibodies. (B-D) Densitometric quantification of mammary tumors shown in panel A for the (B) p66ShcA/Tubulin, (C) p66ShcA/p52ShcA, (D) E-Cadherin/Tubulin and (E) Vimentin/Tubulin ratios. The data is normalized to the parental 4T1-537 tumors. [file 13058_2020_1245_MOESM5_ESM.pdf]

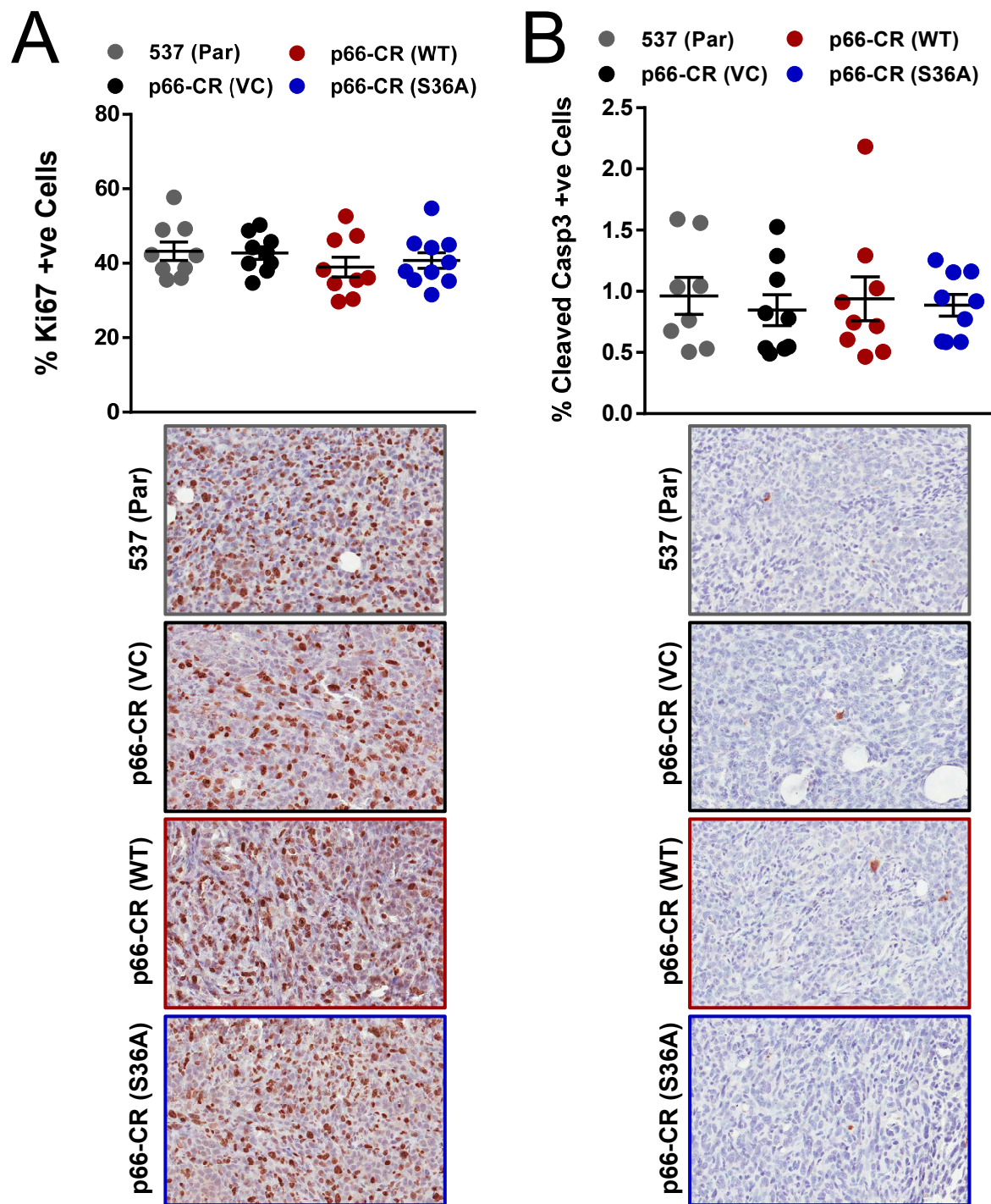

Figure S6

Supplement: Supplementary file 6 — Additional file 6: Figure S6. p66ShcA minimally impacts the growth properties of lung-metastatic triple negative primary breast tumors. Quantification of the percentage (A) Ki67 positive cells and (B) cleaved Caspase-3 positive cells in 4T1-537 parental, p66-CR, p66-CR (WT) and p66-CR (S36A) mammary tumors. The data is shown as positivity ± SEM and is representative of 9–10 tumors per group. Representative images are shown below each graph. [file 13058_2020_1245_MOESM6_ESM.pdf]

Figure S7

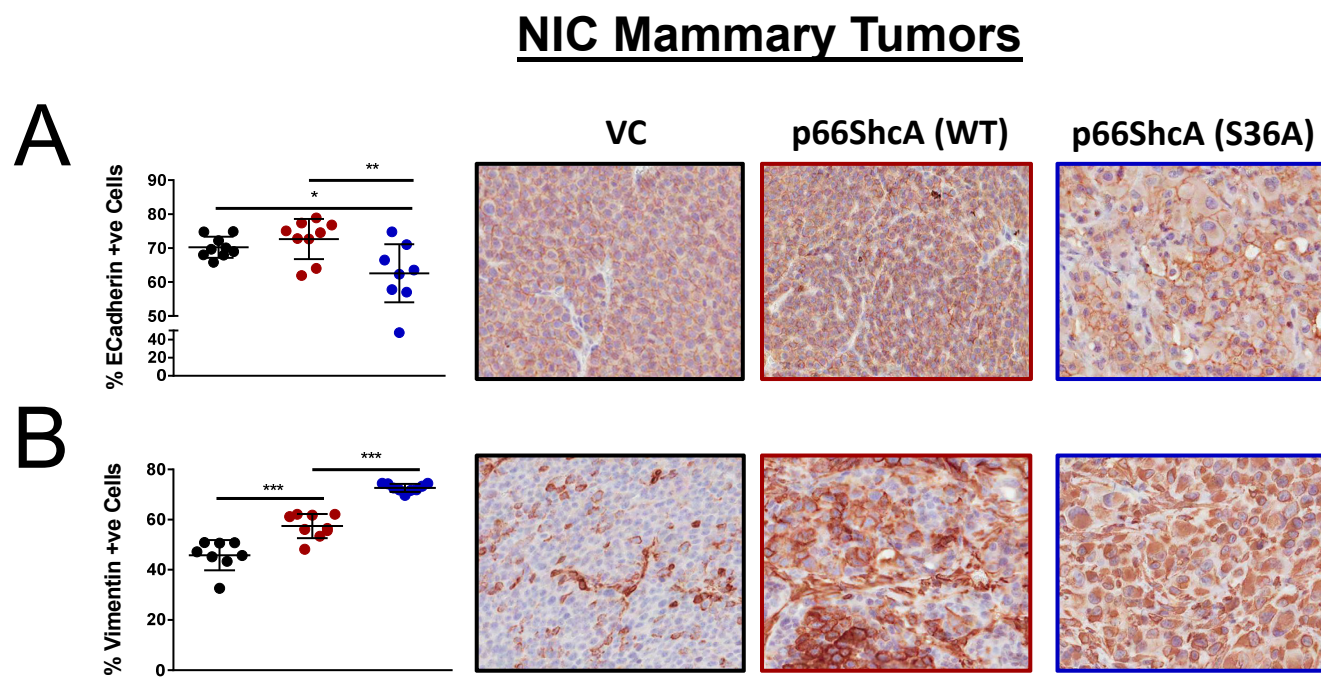

Supplement: Supplementary file 7 — Additional file 7: Figure S7. A p66ShcA-induced epithelial to mesenchymal transition is dispensable for metastatic progression in a luminal breast cancer model. Immunohistochemical analysis of vector control (VC), p66ShcA-WT and p66ShcA-S36A overexpressing NIC mammary tumors using (A) E-Cadherin and (B) Vimentin-specific antibodies. The data is shown as average tumor volume (mm3) ± SEM (n = 7 tumors/group). Representative images are shown. Statistical analysis was performed using a one-way Anova with a Tukey’s multiple comparisons test (*P < 0.05; **P < 0.01; ***P < 0.001; ****P < 0.0001). [file 13058_2020_1245_MOESM7_ESM.pdf]

Figure S8

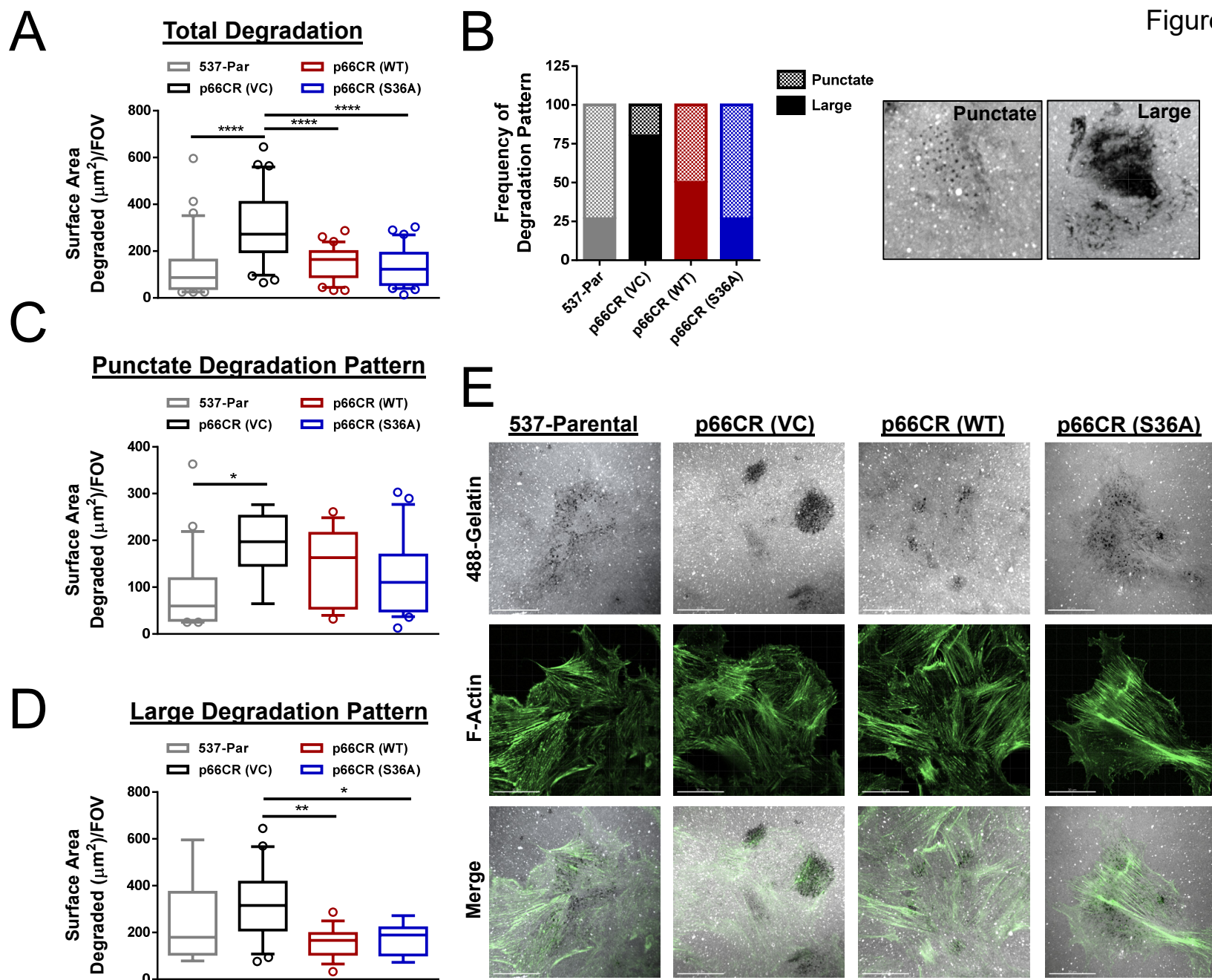

Supplement: Supplementary file 8 — Additional file 8: Figure S8. ShcA p66 is dispensable for invadopodia formation and gelatin degradation. 488 fluorescently-labeled degradation is represented by signal void. (A) Total surface area degraded (μm2) was determined from images taken from three independent experiments (n = 120 FOV, n = 30 each for 4T1-537 (par), p66CR (VC), p66CR (WT) and p66CR (S36A)) and error bars represent s.e.m. (B) Punctate and Large gelatin degradation patches, representative images shown, were classified qualitatively. Frequency of degradation pattern taken from quantified images used for (A). (C) and (D), group the quantified total surface area degraded (μm2) per FOV, from (A), into Punctate and Large degradation patterns, respectively. (E) Representative images of quantified gelatin degradation: F-Actin (green) stained by 647-phalloidin and 488-labelled gelatin (grey). Scale bar is 20 μm in length. Statistical analysis performed using a one-way Anova with a Tukey’s multiple comparisons test (*P < 0.05; **P < 0.01; ****P < 0.0001). [file 13058_2020_1245_MOESM8_ESM.pdf]

Figure S9

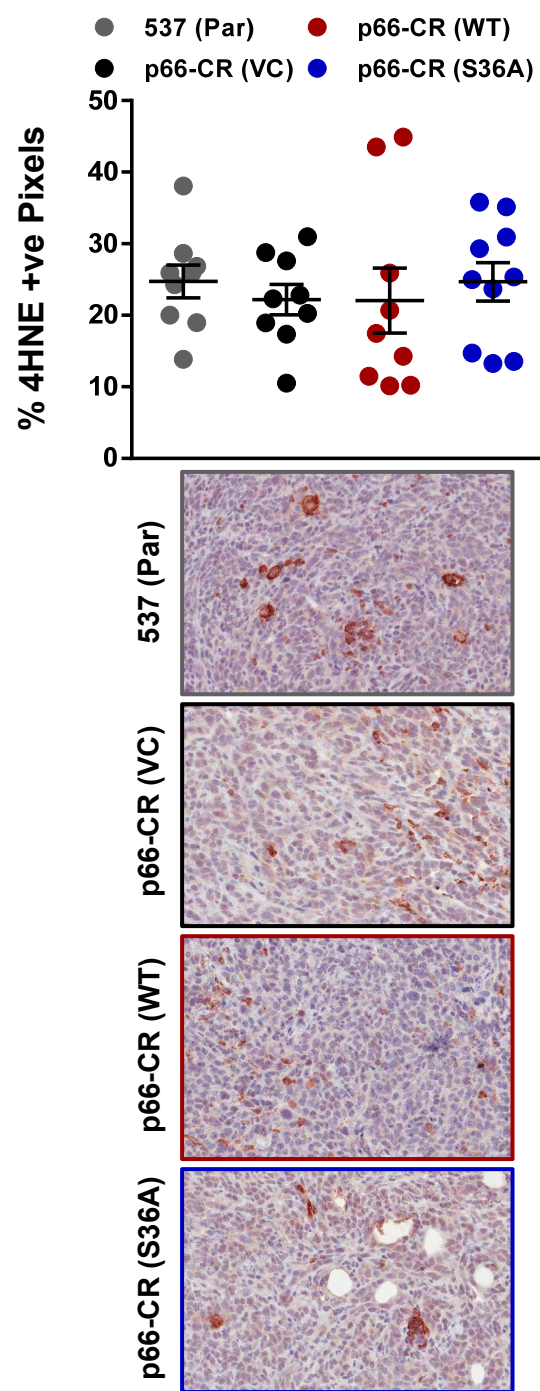

Supplement: Supplementary file 9 — Additional file 9: Figure S9. p66ShcA does not induce oxidative stress in primary tumors in a model of triple negative breast cancer. Quantification of the percentage of 4-HNE positive pixels in 4T1-537 parental, p66-CR, p66-CR (WT) and p66-CR (S36A) mammary tumors. The data is shown as positivity ± SEM and is representative of 9–10 tumors per group. Representative images are shown below each graph. [file 13058_2020_1245_MOESM9_ESM.pdf]

Figure S10

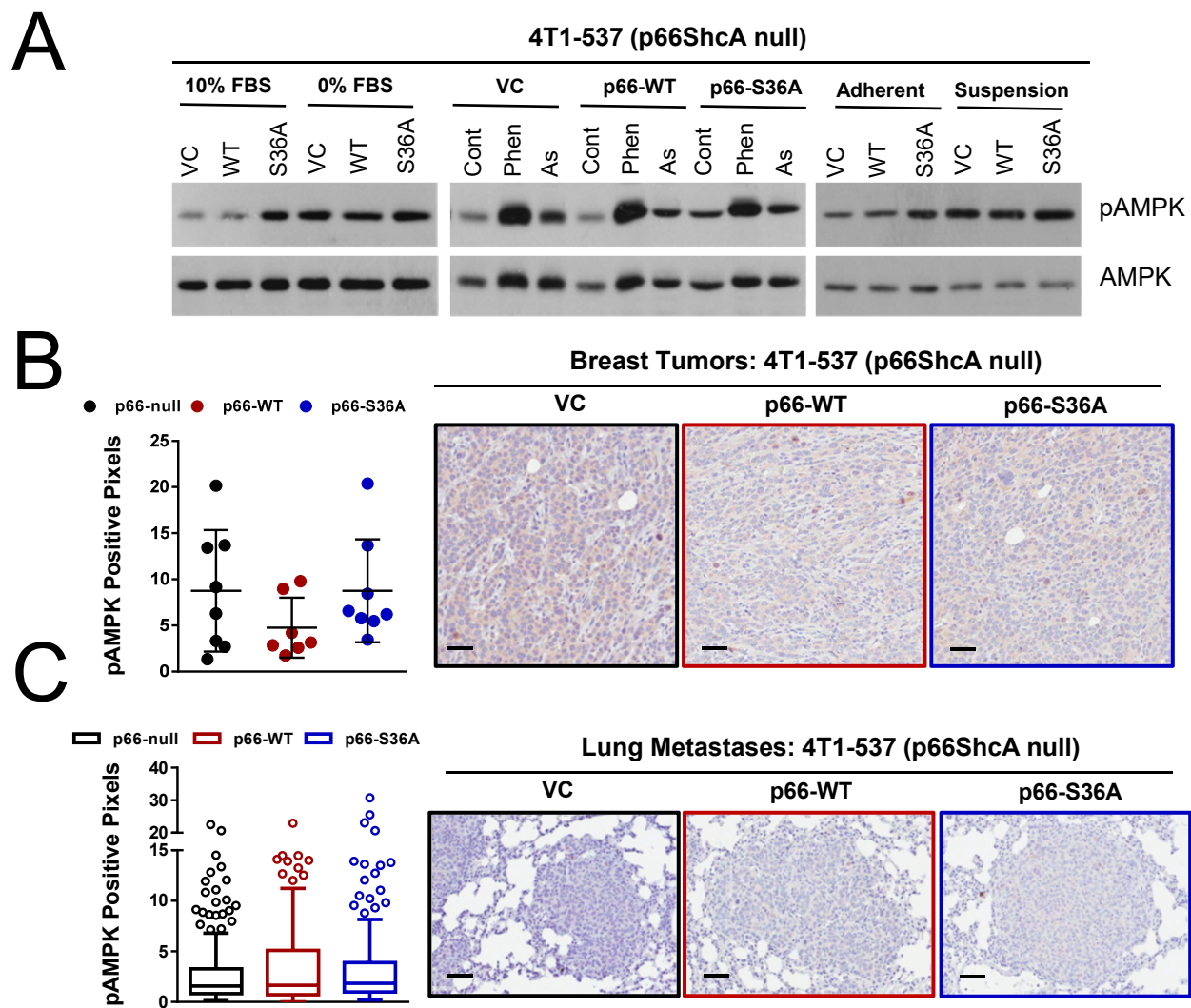

Supplement: Supplementary file 10 — Additional file 10: Figure S10. AMPK activation is not appreciably regulated by p66ShcA in mammary tumors. (A) Whole cell lysates were generated from the indicated cell lines grown under the following conditions: 10% FBS versus 0% FBS for 16 h; 1 mM phenformin for 2 h; 20 μM sodium arsenate for 4 h; suspension cultures on ultra-low attachment plates for 16 h. Immunoblot analysis was performed using pAMPK and AMPK specific antibodies. Percentage of pAMPK positive pixels in primary breast tumors (n = 7–8 tumors/genotype) (B) and in individual lung-metastatic lesions (c) derived from 4T1-537 p66-CR (VC), p66-CR (WT) and p66-CR (S36A) breast cancer cells. For the lung metastases: p66-CR (VC), n = 214 lesions; p66-CR (WT), n = 194 lesions; p66-CR (S36A), n = 202 lesions. (B, C) Representative IHC images for primary breast tumors and lung metastases are shown. [file 13058_2020_1245_MOESM10_ESM.pdf]

Figure S11

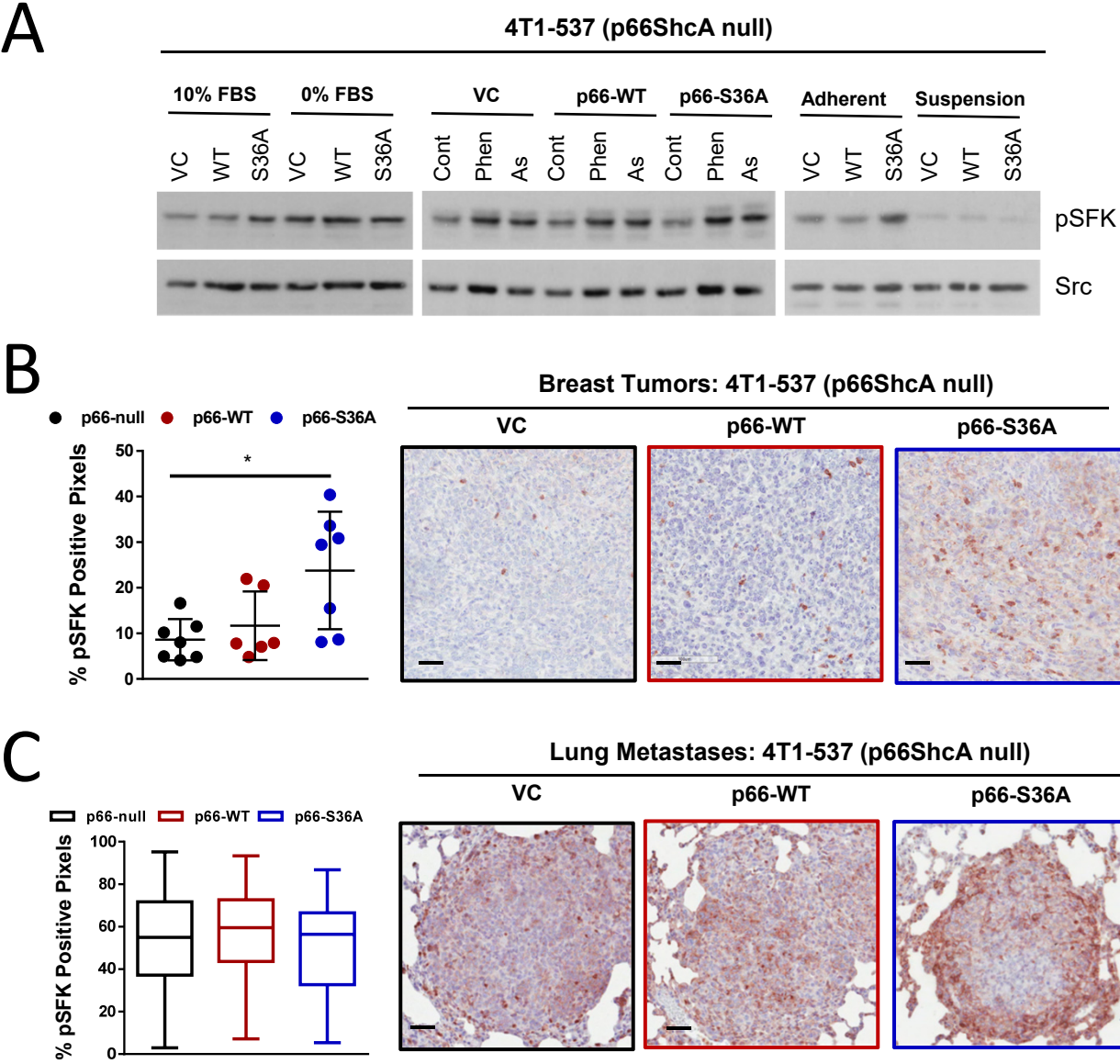

Supplement: Supplementary file 11 — Additional file 11: Figure S11. Src family kinase activation is increased by non-mitochondrial p66ShcA pools in mammary tumors. (A) Whole cell lysates were generated from the indicated cell lines grown under the following conditions: 10% FBS versus 0% FBS for 16 h; 1 mM phenformin for 2 h; 20 μM sodium arsenate for 4 h; suspension cultures on ultra-low attachment plates for 16 h. Immunoblot analysis was performed using pSFK and Src specific antibodies. Percentage of pSFK positive pixels in primary breast tumors (n = 6–7 tumors/genotype) (B) and in individual lung-metastatic lesions (C) derived from 4T1-537 p66-CR (VC), p66-CR (WT) and p66-CR (S36A) breast cancer cells. For the lung metastases: p66-CR (VC), n = 318 lesions; p66-CR (WT), n = 308 lesions; p66-CR (S36A), n = 318 lesions. (B, C) Representative IHC images for primary breast tumors and lung metastases are shown. Statistical analysis performed using a one-way Anova with a Tukey’s multiple comparisons test (*P < 0.05). [file 13058_2020_1245_MOESM11_ESM.pdf]

Figure S12

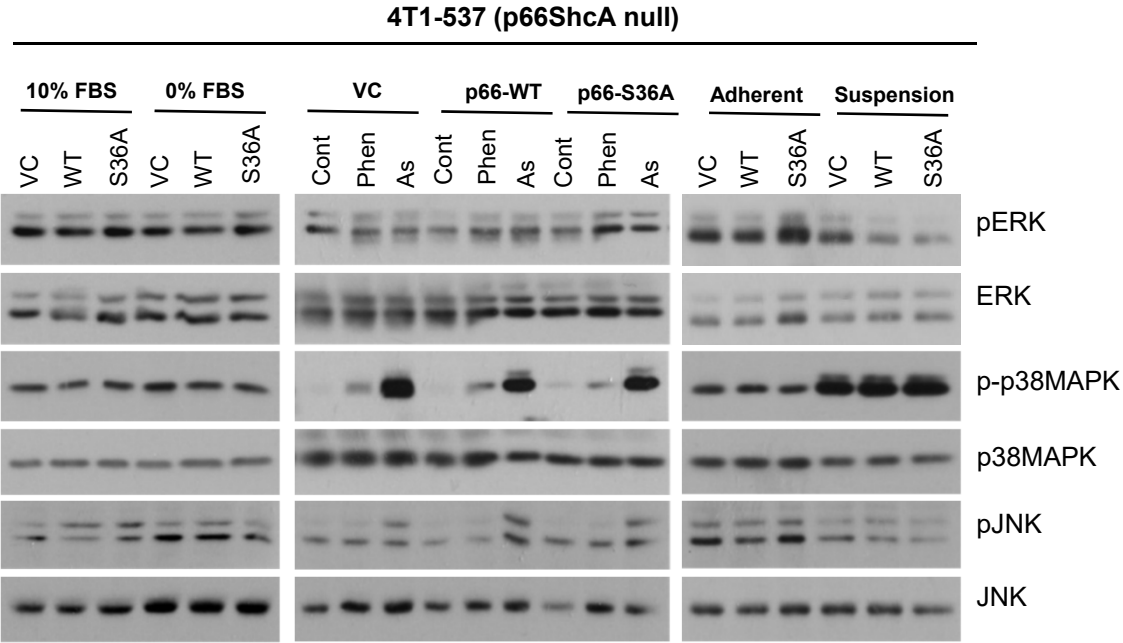

Supplement: Supplementary file 12 — Additional file 12: Figure S12. MAPK family members are not regulated by p66ShcA in lung-metastatic 4T1 breast cancer cells. Whole cell lysates were generated from the indicated cell lines grown under the following conditions: 10% FBS versus 0% FBS for 16 h; 1 mM phenformin for 2 h; 20 μM sodium arsenate for 4 h; suspension cultures on ultra-low attachment plates for 16 h. Immunoblot analysis was performed using phospho-specific and total antibodies against ERK, p38MAPK and JNK. [file 13058_2020_1245_MOESM12_ESM.pdf]
