# Supplementary material for: p66ShcA functions as a contextual promoter of breast cancer metastasis
Source: Breast Cancer Res. 2020 Jan 15;22:7. doi: 10.1186/s13058-020-1245-6 (PMC6964019; doi:10.1186/s13058-020-1245-6)
Supplement: Supplementary file 13 — Additional file 13. Supplemental methods. [file 13058_2020_1245_MOESM13_ESM.pdf]

SUPPLEMENTAL METHODS

Cell Culture

4T1 cells and derivative cell populations were cultured in DMEM medium supplemented with 10% FBS HEPES and 50µg/ml Gentamycin. The NIC tumor cell line was established from a mouse mammary tumor virus/Neu-internal ribosome entry site (IRES)-Cre (NIC) transgenic mouse mammary tumor as described previously (Ursini-Siegel et al., 2008) and cultured in DMEM supplemented with 5% FBS, 100µg/ml penicillin/streptomycin, 50µg/ml Gentamycin#: and Mammary Epithelial Growth Supplement (MEGS; 3ng/ml EGF, 0.5 µg/ml Hydrocortisone, 5µg/ml Insulin, 0.4% v/v Bovine Pituitary Extract).

C-terminally tagged p66ShcA-WT or p66ShcA-S36A cDNAs were subcloned into pMSCV (Clontech), pQCXIB (Addgene) or pQCXIP (Clontech) plasmids. Phoenix (293T) cells were used to produce viral supernatants. Viral infections (16 hours) were enhanced by the addition of 4µg/ml polybrene (Cat. #: TR-1003-G, Cedarlane). Virus containing supernatants were then removed, infected cells rinsed with PBS and subsequently incubated in normal growth medium for 24 hours. Medium was changed to select transduced cells using 2µg/ml puromycin or 10µg/ml blasticidin, as indicated.

CRISPR/Cas9 mediated p66ShcA deletion

Fugene reagent was used to transfect 4T1-537 breast cancer cells with 2µg of guide plasmid (targeting the ShcA CH2 domain – guide sequence: CCACTCCGGAATGAGTCTCTGTC), 2µg of hCas9 and 1µg of pQCXIB (lacking insert or GFP) to confer blasticidin resistance. Resistant cells were then selected using 10µg/ml blasticidin and clonal cell lines were established. Individual

clonal lines were examined for expression of p66ShcA by immunoblot and four that were devoid of p66ShcA expression were pooled into a single polyclonal cell population.

Flow cytometry to quantify reactive oxygen species production

537-4T1 cells were trypsinized, counted and seeded (500,000) onto 6 cm dishes. Cells were allowed to adhere and grow overnight. The following day, cells were stimulated with 10 μM of Actinomycin D (Cat. no.: A1410; Sigma-Aldrich) or DMSO (Cat. no.: DMS666; BioShop) for 2 hours at 37°C under 5% CO_2. Cells were then washed with 1x PBS, trypsinized and centrifuged in the presence of fresh media. Cells were counted, re-suspended (500,000) in fresh media, and stained with MitoSOX^{TM} Red Mitochondrial Superoxide Indicator (Cat. no.: M36008; ThermoFisher Scientific; 1:1,000) for 20 minutes at 37°C and 5% CO_2. Finally, cells were centrifuged, resuspended in 1x PBS with 2% FBS (FACS buffer) and analyzed by flow cytometry. Data were acquired on a FACSCanto II (BD Biosciences) cytometer and analysis was performed using FlowJo software (Tree Star). Exclusion of debris and doublets were applied to all analyses.

Immunofluorescence experiments

537-4T1 cells were trypisinized, counted and seeded (50,000) onto 35 mm cover-glass bottom dishes (Cat. no.: 81158; IBIDI). Cells were allowed to adhere and grow overnight. The following day, cells were pre-treated with 10 μM of Actinomycin D or DMSO for 1 hour at 37°C and 5% CO_2. Cells were then incubated with 50 μM of biotin (Cat. no.: B4501; Sigma-Aldrich) in the presence of Actinomycin D or DMSO for 5 hours at 37°C under 5% CO_2. Finally, cells were fixed with 4% PFA and stained.

Cells were permeabilized with 1% Triton X-100, rinsed with 100 mM glycine in PBS, and blocked with 10% FBS in PBS. Samples were stained with an anti-Tom20 (Cat. no.: sc-11415; Santa Cruz; 1:200) primary antibody for 1 hour at room temperature. Samples were then washed with 0.05% Tween-20/PBS and incubated with Streptavidin Alexa Fluor 488 (Cat. no.: S11223; ThermoFisher Scientific; 1:1,000) and Star 635P goat anti-rabbit (Cat. no: 2-0012-007-2; Abberior; 1:1,000) for 1 hour at room temperature. Finally, samples were washed with 0.05% Tween-20/PBS and stored in PBS for imaging.

Images were acquired on a Leica DMI6000B inverted microscope equipped with a Quorum WaveFx-X1 spinning disk confocal system (Quorum Technologies, Guelph, ON), HCX PL APO 63x/1.40NA oil DIC objective and Prime BSI sCMOS camera (Photometrics, Tucson, AZ). Each cell was illuminated with 491 and 643 nm diode lasers to capture streptavidin and Tom20 signals, respectively. Camera exposure time was set to 5 s. The pinhole size of the spinning disk was fixed at 50 µm.

Images were median filtered 2x2 (ImageJ) and imported into Imaris for further analysis. Cells were manually outlined, and mitochondria were masked using the surfaces function; surface detail was smoothed and set to 0.100 µm with a local background subtraction of 0.200 µm. Surfaces smaller than 10 pixels were removed with filters. To analyze p66ShcA movement from the cytoplasm to mitochondria, a new streptavidin channel was created from the mitochondrial surfaces. The streptavidin signal within the mitochondrial mask was set to 0 so that the sum intensity of the cell did not include contributions from mitochondria. Dividing the sum intensity of the new channel by the total area of the cell provided an estimate of the mean intensity of p66ShcA within the cytoplasm. Finally, the mean intensity of p66ShcA within mitochondria was

divided by the mean intensity of p66ShcA within the cytoplasm to give a measure of localization. Values greater than 1 indicate that there is more p66ShcA localized within mitochondria.

Immunoblot Analysis

Proteins were extracted in PLCγ lysis buffer containing protease and phosphatase inhibitors (5mM NaF, protease inhibitor PIN and 5mM $NaVO_4$). Total proteins were resolved by SDS-PAGE and transferred to Immobilon-P membranes (Cat. #: IPVH00010, Millipore). Membranes were blocked using 5% milk or BSA and then incubated overnight at 4ºC with antibodies against ShcA (Cat. #: 06-203, Millipore), pSer36-p66ShcA (Cat # ab54518, Abcam), pERK (Cat # 9101, Cell Signaling), ERK (Cat # 9102, Cell Signaling), p-p38MAPK (Cat # 9211, Cell Signaling), p38MAPK (Cat # 9212, Cell Signaling), pJNK (Cat # 9251, Cell Signaling), JNK (Cat #9252, Cell Signaling), pAKT (Cat # 4060, Cell Signaling), AKT (Cat # 9272, Cell Signaling), pAMPK (Cat #2535, Cell Signaling), AMPK (Cat # 2532, Cell Signaling), pS65-4EBP1 (Cat #9456, Cell Signaling), 4EBP1 (Cat # 9452, Cell Signaling), p-rS6 (Cat # 2215. Cell Signaling), rS6 (Cat # 2217, Cell Signaling), pSFK (Cat #2101, Cell Signaling), Src (Cat # 2109, Cell Signaling), E-cadherin (Cat. #: 610181, BD Biosciences), Vimentin (Cat. #: ab92547, Abcam), and α-Tubulin (Cat. #: T5168, Sigma-Aldrich). Secondary IgG antibodies coupled to horseradish peroxidase and ECL (Cat. #: 32106, ThermoFisher) were used for protein detection.

Anoikis Assays

The indicated cell cultures were treated for 3 hours with mitomycin (Accord Healthcare) before trypsinizing and resuspending the cells in regular growth medium. Cells were then seeded in regular adherent 6-well plates (Cat. #: 82050-842, VWR) at $5x10^5$ cells/well or ultra low-

attachment 6-well plates (Cat. #: 29443-030, VWR) at $3x10^4$ cells/well and incubated for 24 hours. After incubation, the media from adherent wells were collected, the cells were trypsinized and added to the collected medium. The media from non-adherent wells were collected along with the cells, and 6 wells were combined for each technical replicate. Cells were pelleted by centrifugation and resuspended in 500 µl for the adherent samples, or 200 µl for the non-adherent samples. Cell viability was determined by trypan blue exclusion.

Migration Assays

Boyden chamber assays were performed as described previously (28). Briefly, 100,000 cells were seeded on transwell inserts in serum-free medium and allowed to migrate for 24 hours toward medium containing 10% FBS in the bottom chamber. Cells were then removed from the top of the membrane, and those remaining were fixed with 10% formalin and stained with crystal violet and images captured.

CTC Isolation

Following mammary fat pad injection using the indicated 537 cell populations (puromycin resistant), tumors were allowed to grow until a maximal volume of 2 cm^3, at which point animals were anaesthetized and terminal bleeds collected by intracardiac puncture with a heparin-coated insulin syringe. Circulating tumor cells were isolated from blood as previously described (Ngan et al., 2017). The cells were subsequently resuspended in growth medium and transferred to cell culture dishes coated with 5µg/mL human plasma fibronectin (Cat. #: FC010, Millipore). After 16 hours the medium was changed and supplemented with puromycin to select for CTCs. The cells were cultured for 8 days following seeding, fixed in 10% and then stained with crystal violet.

Scanned images of the wells were imported to ImageJ (v. 1.51j8) and a mask of the colonies was selected by color threshold. Colonies were then counted if larger than 20 pixels^2 and if circularity was greater than 0.2, as defined by the equation $4\pi(\text{area}/\text{perimeter}^2)$.

Gelatin degradation assay and immunofluorescence staining

Gelatin-degradation assays were performed on fluorescently conjugated gelatin-coated 35 mm cover-glass bottom cell culture dishes (FD35-100 WPI). Briefly, sterile 35 mm cell culture dishes were coated with a mix of 0.1 mg ml poly-D-lysine (Cat. #: P6407, Sigma) and 5 mg (cm^2) Fibronectin (Cat. #: FC010, Millipore) in PBS for 20 min, followed by incubation with 0.4% Glutaraldehyde for 10 min. Oregon Green 488 conjugated gelatin (Cat. #: G13186, Invitrogen), was diluted by 1:20 with 0.1% unconjugated gelatin (Cat. #: 07903, Stem Cell Technologies) and used to coat coverslips at 37 °C for 10 min. Coverslips were then incubated with 10 mg ml Sodium Borohydride for 2 min, followed by 70% ethanol for 20 min. Three washes with 1 PBS were performed between each step. DMEM media was added to the coverslips at 37 °C for 1 h before cell plating. 4T1-537 cells were plated (32,000 cells) onto gelatin-coated coverslips and incubated at 37 °C for 24 h. Coverslips were fixed in 4% paraformaldehyde (PFA) and cells were permeabilized with 0.2% Triton X-100, rinsed with 100 mM Glycine in PBS and blocked in 10% FBS in PBS). Atto 647N-Phalloidin (1:1,000; Cat. #: 65906, Sigma-Aldrich) was used.

Image acquisition was performed with ZEN imaging software on a Zeiss LSM710 confocal microscope and a plan-Apochromat 63/1.4 NA oil objective (Carl Zeiss Inc.). Quantification of gelatin area degraded was performed using Imaris v8.3.1 (Bitplane) surfaces function.

REFERENCE

Ngan, E., Stoletov, K., Smith, H. W., Common, J., Muller, W. J., Lewis, J. D., and Siegel, P. M. (2017). LPP is a Src substrate required for invadopodia formation and efficient breast cancer lung metastasis. Nat Commun *8*, 15059.

Ursini-Siegel, J., Hardy, W. R., Zuo, D., Lam, S. H., Sanguin-Gendreau, V., Cardiff, R. D., Pawson, T., and Muller, W. J. (2008). ShcA signalling is essential for tumour progression in mouse models of human breast cancer. EMBO J *27*, 910-920.
